# Treatment of Acute Wound Infections by Degradable Polymer Nanoparticle with a Synergistic Photothermal and Chemodynamic Strategy

**DOI:** 10.1002/advs.202309624

**Published:** 2024-02-26

**Authors:** Fangzhou Chen, Lin Liu, Dongsheng Tang, Hanchen Zhang, Nier Wu, Lin Wang, Hongbo Li, Haihua Xiao, Dongsheng Zhou

**Affiliations:** ^1^ Graduate School Guangzhou Medical University Guangzhou 511436 P. R. China; ^2^ State Key Laboratory of Pathogen and Biosecurity, Beijing Institute of Microbiology and Epidemiology Academy of Military Medical Sciences Beijing 100071 P. R. China; ^3^ Department of Stomatology The First Medical Center Chinese PLA General Hospital Beijing 100853 P. R. China; ^4^ Institute of Chemistry Chinese Academy of Sciences Beijing 100190 P. R. China

**Keywords:** acute wound infections, biodegradable polymer, chemodynamic therapy, ESKAPE pathogens, photothermal therapy

## Abstract

Mild‐heat photothermal antibacterial therapy avoids heat‐induced damage to normal tissues but causes bacterial tolerance. The use of photothermal therapy in synergy with chemodynamic therapy is expected to address this issue. Herein, two pseudo‐conjugated polymers P^M123^ with photothermal units and P^Fc^ with ferrocene (Fc) units are designed to co‐assemble with DSPE‐mPEG_2000_ into nanoparticle NP^M123/Fc^. NP^M123/Fc^ under 1064 nm laser irradiation (NP^M123/Fc^+NIR‐II) generates mild heat and additionally more toxic ∙OH from endogenous H_2_O_2_, displaying a strong synergistic photothermal and chemodynamic effect. NP^M123/Fc^+NIR‐II gives >90% inhibition rates against MDR ESKAPE pathogens in vitro. Metabolomics analysis unveils that NP^M123/Fc^+NIR‐II induces bacterial metabolic dysregulation including inhibited nucleic acid synthesis, disordered energy metabolism, enhanced oxidative stress, and elevated DNA damage. Further, NP^M123/Fc^+NIR‐II possesses >90% bacteriostatic rates at infected wounds in mice, resulting in almost full recovery of infected wounds. Immunodetection and transcriptomics assays disclose that the therapeutic effect is mainly dependent on the inhibition of inflammatory reactions and the promotion of wound healing. What is more, thioketal bonds in NP^M123/Fc^ are susceptible to ROS, making it degradable with highly favorable biosafety in vitro and in vivo. NP^M123/Fc^+NIR‐II with a unique synergistic antibacterial strategy would be much less prone to select bacterial resistance and represent a promising antibiotics‐alternative anti‐infective measure.

## Graphical abstract

NP^M123/Fc^ under 1064 nm laser irradiation (NP^M123/Fc^+NIR‐II) generates mild heat and additionally more toxic ∙OH from endogenous H_2_O_2_, displaying a synergistic effect of PTT and CDT for anti‐infection. Thioketal bonds in NP^M123/Fc^ are susceptible to ROS, making it degradable with highly favorable biosafety. NP^M123/Fc^+NIR‐II representes a biocompatible and broad‐spectrum nanoplatform for antibiotics‐alternative treatment of superficial infections.

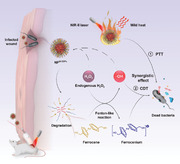

## 1 Introduction

With the wide use and abuse of antibiotics to prevent and treat bacterial infections, antibiotic resistance is occurring increasingly. Particularly, ESKAPE pathogens including *Enterococcus faecium* (*Ec. faecium*), *Staphylococcus aureus* (*S. aureus*), *Klebsiella pneumoniae* (*K. pneumoniae*), *Acinetobacter baumannii* (*A. baumannii*), *Pseudomonas aeruginosa* (*P. aeruginosa*), and *Enterobacter* species (especially *Eb. cloacae* and *Eb. hormaechei*) are commonly characteristic of multidrug resistance (MDR), and they represent the leading cause of refractor infections in hospital settings throughout the world, leading to higher medical costs, prolonged hospital stays, and increased mortality.^[^ ^1^ ^]^

Contemporary antibiotics‐alternative therapeutic strategies such as nanotechnology are potential options for combating MDR bacteria including ESKAPE pathogens.^[^ ^2^ ^]^ Various organic or inorganic nanomaterials have the ability to evade existing mechanisms of acquired drug resistance, taking advantages such as bare selection for resistance, minimal invasion, and low host toxicity.^[^ ^3^ ^]^ In addition, the unique physico–chemical properties (such as size, shape, and surface chemistry) of nanomaterials render the elevated targeting attack pathogens.^[^ ^3^ ^]^ Currently, nanotechnology‐based antibacterial agents mainly rely on four major principles of action, namely sonodynamic therapy (SDT), photodynamic therapy (PDT), chemodynamic therapy (CDT), and photothermal therapy (PTT). SDT utilizes senosensitizers to produce reactive oxygen species (ROS) from surrounding oxygen under low‐intensity ultrasound, killing bacteria through the synergistic action of ROS and ultrasound.^[^ ^4^ ^]^ Likewise, PDT employs photosensitizers that can transfer photon energy to surrounding oxygen to produce ROS.^[^ ^5^ ^]^

PTT makes use of photothermal agents to absorb energy from the near‐infrared region I and II (NIR‐I and NIR‐II) light for the production of heat.^[^ ^6^, ^7^, ^8^ ^]^ However, a high local temperature of up to 70 °C is required to kill bacteria completely if PTT is used alone.^[^ ^9^ ^]^ This will bring pain to the patients and even damage to surrounding tissues.^[^ ^9^ ^]^ On the contrary, the use of mild heat (commonly ≤ 48 °C in vivo) in PTT can avoid heat‐induced damage of normal tissues,^[^ ^10^ ^]^ but mild heat will lead to bacterial tolerance due to inducible expression of heat shock proteins.^[^ ^11^ ^]^ Combining PTT with another different strategy (e.g., CDT,^[^ ^12^ ^]^ PDT,^[^ ^13^ ^]^ or quaternary phosphonium^[^ ^14^ ^]^) could address the above issues and, in this case, CDT‐generated hydroxyl radicals (∙OH), PDT‐generated ROS or cationic quaternary phosphonium agent acts as a synergistic factor of PTT‐generated mild heat, endowing improved broad‐spectrum antibacterial effects.

CDT is applied to convert hydrogen peroxide (H_2_O_2_) into more toxic ∙OH via a Fenton or Fenton‐like reaction.^[^ ^15^ ^]^ In this context, Ferrocene (Fc) has attracted the interest in developing nanomaterials to convert endogenous abundant H_2_O_2_ at infection sites to participate in Fenton‐like reaction, where H_2_O_2_ oxidizes Fc into ferrocenium (Fce) and meanwhile itself is catalyzed into ∙OH.^[^ ^16^, ^17^, ^18^ ^]^

**Scheme 1:**
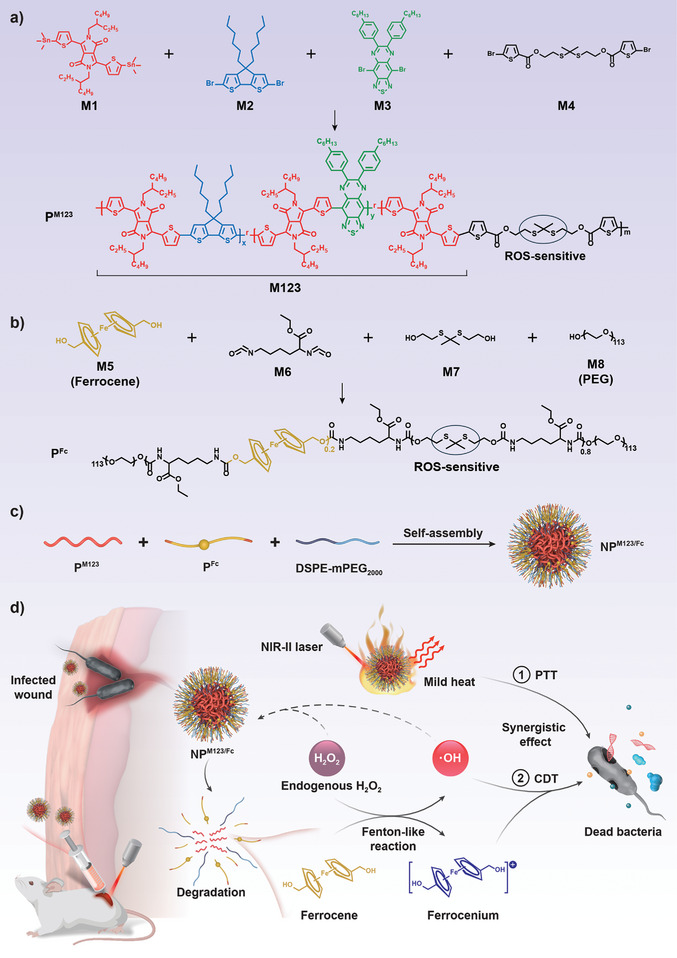
Schematic illustration of development of NP^M123/Fc^ for treatment of acute wound infections. a) The polymer P^M123^ was synthesized from the four monomers namely M1, M2, M3, and M4, among which M1, M2, M3 constituted the photothermal agent M123 while M4 contained the ROS‐sensitive thioketal bond. b) The polymer P^Fc^ was synthesized from another four monomers namely M5 (Fc), M6 (containing thioketal bond), M7, and M8 (mPEG). c) P^M123^, P^Fc^, and DSPE‐mPEG_2000_ were self‐assembled together into nanoparticle NP^M123/Fc^. d) NP^M123/Fc^ under 1064 nm laser irradiation (NP^M123/Fc^+NIR‐II) generates mild heat and additionally more toxic ∙OH from endogenous H_2_O_2_, displaying a synergistic effect of PTT and CDT for anti‐infection. The thioketal bonds in NP^M123/Fc^ are susceptible to ROS, making it degradable with highly favorable biosafety in vitro and in vivo. NP^M123/Fc^+NIR‐II represented a biocompatible and broad‐spectrum nanoplatform for antibiotics‐alternative treatment of superficial infections.

Herein, we developed two biodegradable polymers namely P^M123^ and P^Fc^, which were assembled with commercial DSPE‐mPEG_2000_ to generate the nanoparticle NP^M123/Fc^ (**Scheme** **1**). P^M123^ contained the NIR‐II polymer photothermal agent M123 plus the ROS‐sensitive thioketal bonds, while P^Fc^ harbored Fc as well as the thioketal bonds. NP^M123/Fc^ under 1064 nm laser irradiation (NP^M123/Fc^+NIR‐II) exhibited M123‐mediated photothermal activity and Fc‐dependent chemodynamic effect, which worked in a synergistic manner. NP^M123/Fc^+NIR‐II was highly efficient in inhibiting MDR ESKAPE pathogens in vitro and in treating infected wounds in a mouse model. NP^M123/Fc^ was degradable upon ROS, and it would be highly degradable, rapidly cleared, and hypotoxic in the body. NP^M123/Fc^+NIR‐II with a unique two‐way synergistic antibacterial strategy would be much less prone to select bacterial resistance and represented a promising measure for antibiotics‐alternative therapy.

## 2 Results and Discussion

### 2.1 Preparation and Characterization of Polymers and Nanoparticles

The present study involved three different polymers (i.e., P^M123^, P^Fc^, and DSPE‐mPEG_2000_) and three distinct nanoparticles (i.e., NP^M123^, NP^Fc^, and NP^M123/Fc^) as shown in Scheme 1 and Scheme S1 (Supporting Information). P^M123^ (Scheme 1a) was synthesized from the four monomers namely M1, M2, M3, and M4. M1 was the linker responsible for linking M2, M3 and M4 to form P^M123^. M2 mainly endowed P^M123^ with photothermal properties. M3 caused the UV absorption wavelength of P^M123^ to be redshifted and thus made P^M123^ excitable at 1064 nm. The M123 moiety as a whole could be recognized as a polymer photothermal agent. M4 contained the ROS‐sensitive thioketal bond, conferring the degradability of P^M123^. P^Fc^ (Scheme 1b) was synthesized from another four monomers namely M5, M6, M7, and M8. M5 was Fc. M6 was the linker responsible for linking M5, M7, and M8 to form P^Fc^. M7 contained the ROS‐sensitive thioketal bond, conferring the degradability to P^Fc^. Since M5, M6, and M7 were all hydrophobic, M8 (mPEG) was added to render the amphipathicity of P^Fc^. The successful synthesis of P^M123^ (Figure S1, Supporting Information) or P^Fc^ (Figure S2, Supporting Information) was validated by ^1^H NMR (nuclear magnetic resonance) spectra. For P^M123^, the peaks at 0.5 to 2 ppm could be attributed to the alkane chain, the peaks at 7 to 9.5 ppm could be attributed to the hydrogen of thiophene, and the peaks at 2.5 to 4.5 ppm could be attributed to the methylene group of ROS‐sensitive thioketal bond (Figure S1, Supporting Information). For P^Fc^, the peaks at 7 to 8 ppm belonged to Fc and the peak at 1.5 ppm belonged to the methyl of thioketal bond (Figure S2, Supporting Information), indicating that P^Fc^ contained Fc and ROS‐sensitive moieties. Hydrophobic P^M123^ or amphipathic P^Fc^ was subsequently self‐assembled with amphipathic DSPE‐mPEG_2000_, generating NP^M123^ or NP^Fc^, respectively (Scheme S1, Supporting Information). P^M123^, P^Fc^, and DSPE‐mPEG_2000_ were self‐assembled together into NP^M123/Fc^ (Scheme 1c).

**Figure 1:**
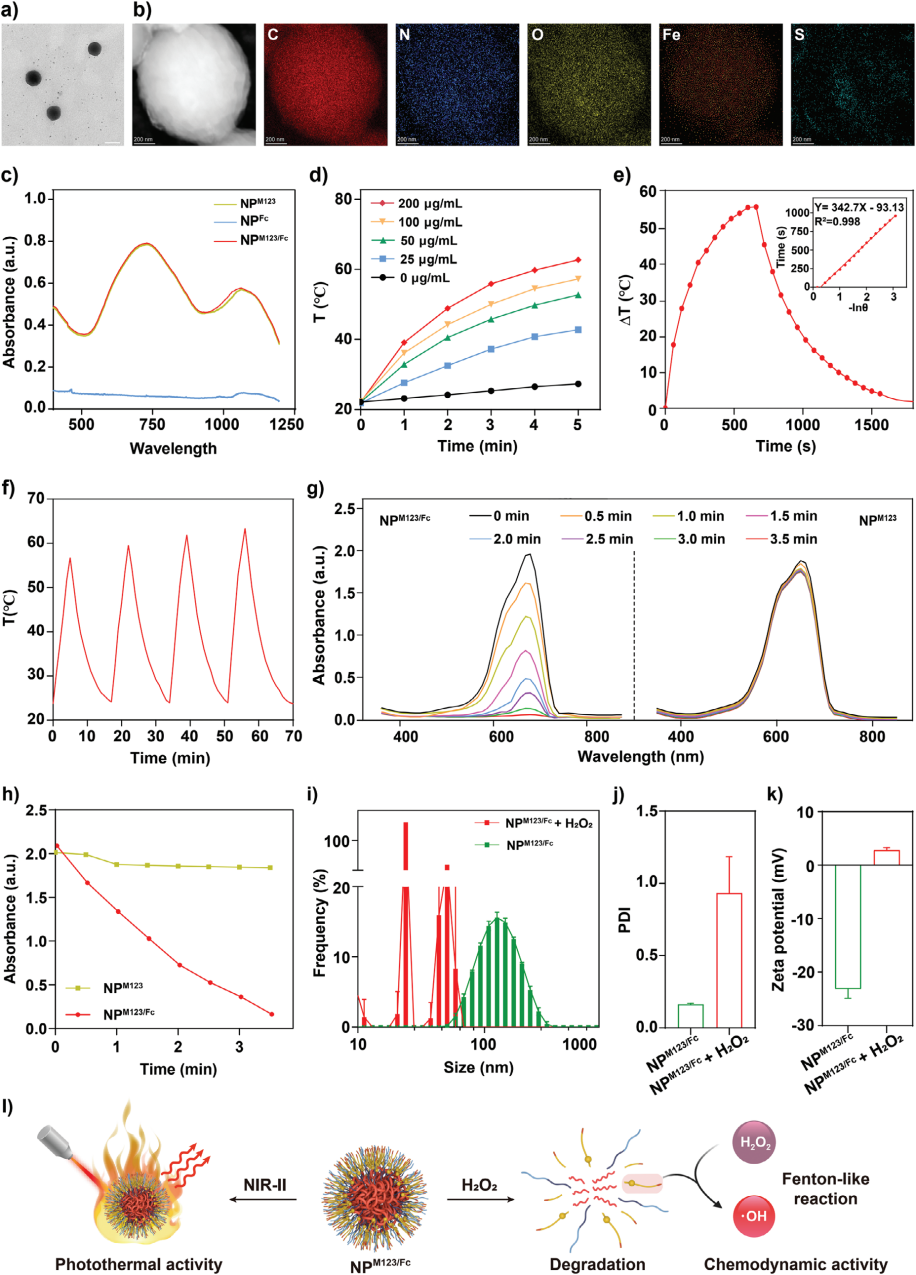
Characterization of NP^M123/Fc^. a) TEM images of NP^M123/Fc^. Scale bar is 100 nm. b) STEM and EDXS images of NP^M123/Fc^. Scale bar is 200 nm. c) UV–vis–NIR absorption spectra of NP^M123^, NP^Fc^, and NP^M123/Fc^. d) Time*–*concentration*–*temperature curves of NP^M123/Fc^ under 1064 nm laser irradiation (1.0 W cm^−2^, 5 min). e) Time‐dependent heating and cooling curve of 100 µg mL^−1^ NP^M123/Fc^ under 1064 nm laser irradiation (1.0 W cm^−2^, 11 min). The 𝜂 value was calculated, through linear regression, from this heating and cooling curve. f) Time*–*temperature curve of four cycles of heating and cooling of NP^M123/Fc^ under 1064 nm laser irradiation (1.0 W cm^−2^, 5 min). g) Absorption curves of MB as a selective trapping agent to detect ∙OH in the reaction mixture of NP^M123/Fc^+H_2_O_2_ or NP^M123^+H_2_O_2_ after different incubation times. h) Trends of UV absorption at 410 nm as indicated by the above MB‐based detection results. DLS detection of i) diameter sizes, j) PDIs, and k) average Zeta potentials of NP^M123/Fc^ in the presence and absence of H_2_O_2_. l) Schematic illustration of H_2_O_2_‐mediated degradability, M123‐dependent NIR‐II photothermal activity, and Fc‐dependent chemodynamic activity of NP^M123/Fc^ in vitro.

As determined by transmission electron microscopy (TEM) and scanning electron microscopy (SEM), NP^M123^, NP^Fc^, and NP^M123/Fc^ displayed uniform spherical morphology with average diameters at about 106, 108, and 120 nm, respectively (**Figure** **1**a; Figures S3 and S4, Supporting Information). Energy dispersive X‐ray spectral scanning (EDXS) showed that there were the key elements C, N, O, Fe and S in NP^M123/Fc^ (Figure 1b), and the presence of Fe confirmed the successful doping of Fc into NP^M123/Fc^.

UV*–*vis*–*NIR absorption spectra denoted that each of NP^M123^ and NP^M123/Fc^ had two major absorption peaks at 740 and 1064 nm, indicating that these nanoparticles had absorption extended in NIR‐II (Figure 1c). In order to investigate the photothermal properties, NP^M123^ or NP^M123/Fc^ at 0 to 200 µg mL^−1^ in the aqueous solution was irradiated with a 1064 nm laser at 1 W cm^−2^ for 5 min, and the time‐temperature curves were recorded (Figure 1d; Figure S5, Supporting Information). The heating process became faster with the increasing of NP^M123/Fc^ concentrations, and 100 µg mL^−1^ NP^M123/Fc^ (used for the following in vitro and in vivo antibacterial assays) gave a temperature increase to 50 °C under laser irradiation for 3 min (Figure 1d; Figure S5, Supporting Information). NP^M123^ showed similar results (Figure S6, Supporting Information). The photothermal conversion efficiency (𝜂) of NP^M123^ and NP^M123/Fc^ were calculated as 38.16% and 37.8% respectively (Figure 1e; Figure S7, Supporting Information), which were comparable to the majority of reported polymer‐based NIR‐II photothermal nanomaterials.^[^ ^19^, ^20^ ^]^ After four cycles of heating (irradiation) and cooling, 100 µg mL^−1^ NP^M123/Fc^ gave the nearly same temperature elevation to about 60 °C in each cycle and the almost constant cooling rates at about 3.2 °C min^−1^ for all cycles (Figure 1f). NP^M123^ showed similar results (Figure S8, Supporting Information). Therefore, NP^M123/Fc^ containing the photothermal agent M123 had an efficient and stable NIR‐II photothermal effect in vitro upon 1064 nm laser irradiation.

Given the participation of Fc and H_2_O_2_ in a Fenton‐like reaction,^[^ ^16^, ^17^, ^18^ ^]^ 100 µg mL^−1^ NP^M123^ or NP^M123/Fc^ was mixed with 1 mm H_2_O_2_ and 2 mm 1,3‐diphenylisobenzofuran (DPBF), followed by incubation for 0–3.5 min, to test consumption of H_2_O_2_ (Figures S9 and S10, Supporting Information). DPBF with an absorption peak at 410 nm was used herein as a selective trapping agent of ROS including H_2_O_2_ and ∙OH.^[^ ^21^ ^]^ With the prolonging of incubation times, the absorption peaks as detected by SpectraMax Microplate Reader were decreased for NP^M123/Fc^, NP^M123^ and NP^Fc^ (Figure S9, Supporting Information), but the absorbance of NP^M123/Fc^ or NP^Fc^ at 410 nm decreased faster than that of NP^M123^ (Figure S10, Supporting Information); therefore, both NP^M123/Fc^ and NP^Fc^ (but not NP^M123^) would produce other ROS from H_2_O_2_, and Fc played a role in ROS production in NP^M123/Fc^. To test the production of ∙OH from H_2_O_2_, the ∙OH‐specific trapping agent methylene blue (MB) was used to determine its absorption peak at 664 nm; with the prolonging of incubation times, NP^M123/Fc^ or NP^Fc^ gave rapidly decreased absorption peaks, but NP^M123^ gave almost unchanged absorption peaks (Figure 1g; Figure S11, Supporting Information). Moreover, the absorbance of NP^M123/Fc^ or NP^Fc^ at 664 nm decreased faster than that of NP^M123^ (Figure 1h; Figure S11, Supporting Information). These results indicated that NP^M123/Fc^ or NP^Fc^ (but not NP^M123^) produced ∙OH from H_2_O_2_, which was dependent on Fc. The Fc release kinetics of NP^M123/Fc^ in vitro was measured by ICP‐MS‐based detection of dissociated Fc; as shown in Figure S12 (Supporting Information), there was a gradual increase in Fc release from 0 to 48 h whether there was the presence of 10 mm H_2_O_2_ or not, but the detected Fc release rate 76.48% in the presence of 10 mm H_2_O_2_ at 48 h was much higher the counterpart value (23.31%) in the presence of PBS. Therefore, Fc could be effectively released from NP^M123/Fc^ in the presence of H_2_O_2_. To test the oxidation of Fc, 1 mg mL^−1^ P^Fc^ was incubated with 1 mm H_2_O_2_ at 37 °C for 24 h, and then X‐ray photoelectron spectroscopy (XPS) revealed that the addition of H_2_O_2_ made the change of iron valence state from +2 into +3 (Figure S13, Supporting Information). Therefore, NP^M123/Fc^ had the chemodynamic effect in vitro to produce ∙OH from H_2_O_2_ and meanwhile to oxidize Fc into Fce.

To test sensitivity to H_2_O_2_ in vitro, NP^M123^, NP^Fc^, and NP^M123/Fc^ were incubated with 1 mm H_2_O_2_ for 3.5 min. TEM and SEM disclosed nearly complete disintegration of nanoparticles (Figures S14 and S15, Supporting Information). As determined by dynamic light scattering (DLS), the average diameter sizes (Figure 1i) and the polydispersity indexes (PDIs, Figure 1j) of NP^M123/Fc^ were changed from 123.07 to 2531.33 nm (giving a huge fold increase of 20.57) and from 0.16 to 0.93 (approaching nearly 1^[^ ^22^ ^]^), respectively, and moreover the average Zeta potentials of NP^M123/Fc^ were changed from −31.08 mV to + 7.22 mV (Figure 1k). In addition, NP^M123^ and NP^Fc^ gave similar DLS‐detected results (Figure S16, Supporting Information). Therefore, the ROS‐sensitive thioketal bonds in NP^M123^, NP^Fc^, and NP^M123/Fc^ were degradable upon H_2_O_2_ in vitro and then these nanoparticles were disintegrated in the solutions; as the degradation of NP^Fc^ or NP^M123/Fc^, Fc was released in the solution and then oxidized by H_2_O_2_ into positively charged Fce, thereby causing the change in overall Zeta potentials from negative charge into positive. In addition, the degradation of NP^M123^ or NP^M123/Fc^ was along with the release of photothermal agent M123.

Taken together, as determined by comprehensive in vitro characterizations, NP^M123/Fc^ was degradable upon H_2_O_2_ and, moreover, it exhibited the photothermal activity to produce mild heat from NIR‐II laser energy and also the Fc‐dependent chemodynamic activity to produce ∙OH from H_2_O_2_ (Figure 1l).

### 2.2 Antibacterial Efficacy Against ESKAPE Pathogens In Vitro

**Figure 2:**
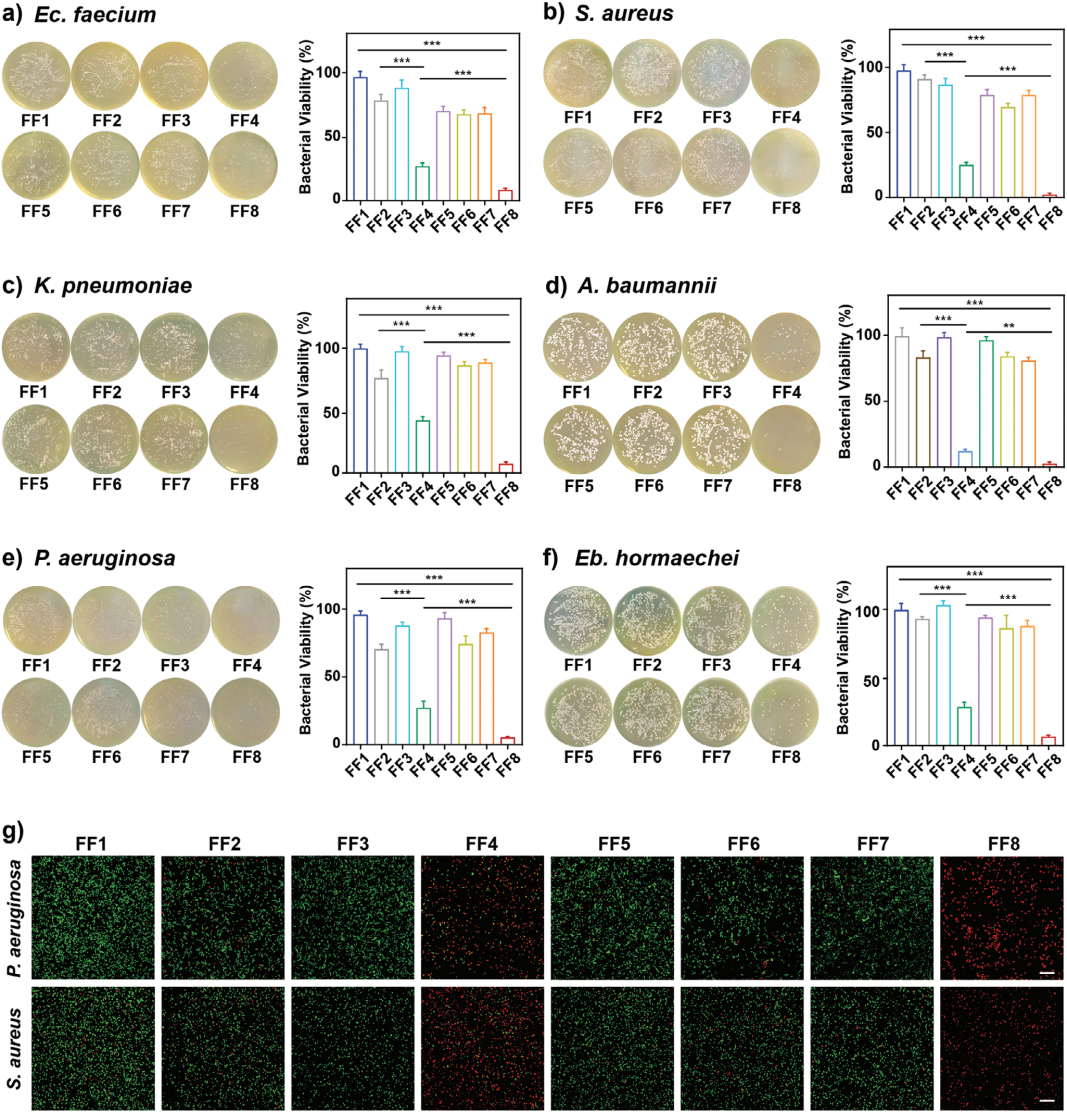
Antibacterial efficacy against ESKAPE pathogens in vitro. FF1: Mock, FF2: NIR‐II, FF3: NP^M123^, FF4: NP^M123^+NIR‐II, FF5: NP^Fc^, FF6: NP^Fc^+NIR‐II, FF7: NP^M123/Fc^, FF8: NP^M123/Fc^+NIR‐II. NIR‐II represents 1064 nm laser irradiation (1.0 W cm^−2^, 3.5 min). a–f) Representative photos of plate count agars for ESKAPE pathogens post treatment, and corresponding statistical analysis of bacterial counts. Data are presented as mean ± SD (*n* = 6). ns: *p* ≥ 0.05, *: *p* < 0.05, **: *p* < 0.01, ***: *p* < 0.001. g) Representative CLSM images (*n* = 3) of live/dead bacteria post treatment. Red and green fluorescence signals correspond to dead and live bacterial cells, respectively. Scale bar is 100 µm. 1 mm H_2_O_2_ are added for all experiments.

The in vitro antibacterial efficacies of NP^M123^, NP^Fc^, and NP^M123/Fc^ were measured by using plate counting method with clinical MDR ESKAPE isolates as model pathogens, and a total of eight treatment groups namely FF1 (Mock), FF2 (NIR‐II), FF3 (NP^M123^), FF4 (NP^M123^+NIR‐II), FF5 (NP^Fc^), FF6 (NP^Fc^+NIR‐II), and FF7 (NP^M123/Fc^), and FF8 (NP^M123/Fc^+NIR‐II) was set (**Figure** **2**). 1 mm H_2_O_2_ was added for all in vitro antibacterial experiments, which was needed to degrade NP^M123^, NP^Fc^, and NP^M123/Fc^ to release M123 or/and Fc for subsequent bactericidal actions. No significant inhibition effects on all six ESKAPE bacteria (Figure 2a–f) were found for the five scheduled control groups FF1, FF2, FF3, FF5, and FF7, confirming that each nanoparticle along with 1 mm exogenous H_2_O_2_ had very limited bactericidal activity in the absence of laser irradiation. FF4 (PTT alone) had a higher bactericidal activity relative to FF6 (CDT alone); notably, when PTT and CDT worked together (FF8), the highest antibacterial efficacy against both Gram‐positive (Figure 2a,b) and Gram‐negative (Figure 2c–f) ESKAPE bacteria were detected with inhibition rates at 97.79%, 91.42%, 92.82%, 97.6%, 94.54%, and 93.53% for *Ec. faecium*, *S. aureus*, *K. pneumoniae*, *A. baumannii*, *P. aeruginosa*, and *Eb. hormaechei*, respectively.

*P. aeruginosa* and *S. aureus* were then selected as the representatives of Gram‐negative and Gram‐positive ESKAPE pathogens, respectively, to investigate whether FF8 had the PTT/CDT synergistic effect to combat bacteria. First, the synergistic factor Q values (see Supporting Information for computational formula and evaluation criterion) were calculated as 1.24 and 1.23 for *P. aeruginosa* and *S. aureus*, respectively. These two Q values were larger than 1.15 which was commonly used as a cutoff indicator of “strong synergy.”^[^ ^23^ ^]^ Second, staining with live/dead double fluorescent dyes SYTO‐9/propidium iodide (PI) and imaging with confocal laser scanning microscopy (CLSM) were carried out and the results indicated that, for either *P. aeruginosa* or *S. aureus* (Figure 2g), a few red fluorescences (dead) and abundant green fluorescence (live) were observed for bacterial cells treated with FF6, while abundant red fluorescence (dead) and a few green fluorescence (live) were observed for bacterial cells treated with FF4; by contrast, nearly all bacterial cells treated with FF8 gave red fluorescence (dead), further confirming the synergy.

Taken together, NP^M123/Fc^ employed 1064 nm laser‐triggered photothermal effect and Fc‐dependent chemodynamic effect in a synergistic manner, achieving the excellent bactericidal efficacy against MDR ESKAPE pathogens in vitro.

### 2.3 Bacterial Morphological and Biochemical View of Antibacterial Mechanism In Vitro

**Figure 3:**
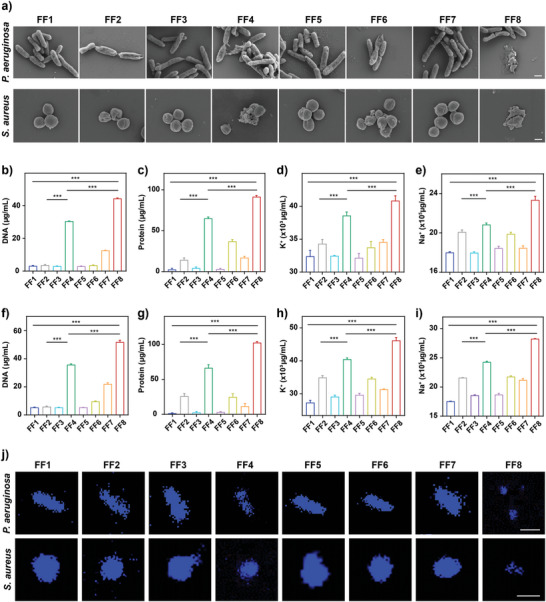
Bacterial morphological and biochemical view of antibacterial mechanism in vitro. FF1: Mock, FF2: NIR‐II, FF3: NP^M123^, FF4: NP^M123^+NIR‐II, FF5: NP^Fc^, FF6: NP^Fc^+NIR‐II, FF7: NP^M123/Fc^, FF8: NP^M123/Fc^+NIR‐II. NIR‐II represents 1064 nm laser irradiation (1.0 W cm^−2^, 3.5 min). a) SEM images of *P. aeruginosa* and *S. aureus* post treatment. Scale bar is 500 nm. Leakage of b) DNA, c) protein, d) K^+^, and e) Na^+^ by *P. aeruginosa* post treatment. Leakage of f) DNA, g) protein, h) K^+^, and i) Na^+^ by *S. aureus* post treatment. j) CLSM images of DAPI‐stained bacterial DNAs post treatment. Scale bar is 1 µm. Data are presented as mean ± SD (*n* = 6). ns: *p* ≥ 0.05, *: *p* < 0.05, **: *p* < 0.01, ***: *p* < 0.001. 1 mm H_2_O_2_ are added for all experiments.

The underlying mechanism of antibacterial action in vitro was explored with respective to bacterial morphological and biochemical changes using MDR *S. aureus* and *P. aeruginosa* as representatives by setting the abovementioned eight treatment groups (**Figure** **3**). First, SEM (Figure 3a) and TEM (Figure S17, Supporting Information) was employed to observe the integrity of bacterial morphology and cell membrane after treatment: i) NP^M123/Fc^+NIR‐II resulted in very severe morphological wrinkling and cell membrane disruption; ii) NP^M123^+NIR‐II induced relatively lower levels of wrinkling and disruption; and iii) only slight wrinkling and concavity on bacterial cell surfaces was observed for the those treated with NIR‐II and no changes could be observed for those treated with the remaining 5 treatment groups. The damage to bacterial cell membrane by nanoparticles would lead to the leakage of bacterial cytoplasmic components.^[^ ^23^ ^]^ Second, the contents of DNA, proteins, K^+^, and Na^+^ in the supernatants of *P. aeruginosa* (Figure 3b–e) and *S. aureus* (Figure 3f–i) suspensions after treatment. The results showed that the largest amounts of DNA, proteins, K^+^, and Na^+^ were detected for those treated with NP^M123/Fc^+NIR‐II, denoting that NP^M123/Fc^+NIR‐II resulted in the most severe bacterial damage. Third, CLSM (Figure 3j) was utilized to characterize DNA damage by staining bacterial DNA with 4′,6‐diamidino‐2‐phenylindole (DAPI) dye with blue fluorescence: i) only a little fluorescence intensity was observed for those treated with NP^M123/Fc^+NIR‐II, indicating the occurrence of most severe DNA damage; and ii) bright fluorescence could be observed for all the other treatment groups, among which the fluorescence intensity of those treated with NP^M123^+NIR‐II was much lower than the remaining six treatment groups. Taken together, NP^M123/Fc^+NIR‐II exhibited the best efficacy in damaging bacterial cell membranes (causing leakage of cytoplasmic components) and meanwhile inducing bacterial DNA damage.

### 2.4 Bacterial Metabolomic View of Antibacterial Mechanism In Vitro

**Figure 4:**
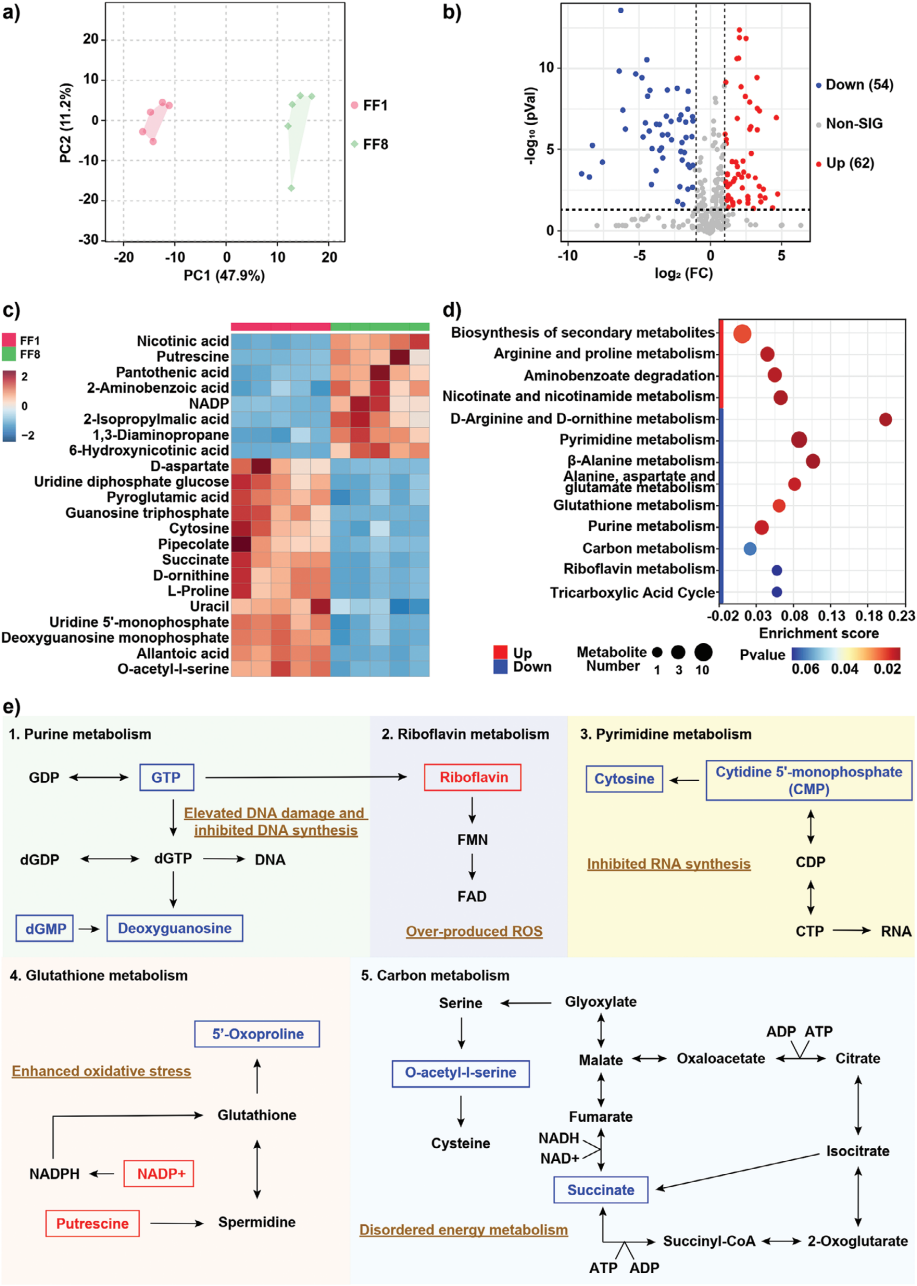
Metabolomics analysis of *P. aeruginosa* after treatment. a) PCA plot of clustered datasets between the two treatment groups NP^M123/Fc^+NIR‐II (FF8) and Mock (FF1). NIR‐II represents 1064 nm laser irradiation (1.0 W cm^−2^, 3.5 min). b) Volcano plot of DRMs identified for NP^M123/Fc^+NIR‐II compared to Mock; Up: Up‐regulated metabolites. Down: Down‐regulated metabolites. c) Heatmaps of major DRMs. d) KEGG pathway enrichment analysis of DRMs. e) Diagrams of major DRMs and related pathways. Red represents up‐regulation and blue stands for down‐regulation. Experiments were performed with five independent biological replicates. 1 mm H_2_O_2_ are added for all experiments.

To characterize bacterial metabolomic response after treatment in vitro, a metabolomics analysis was performed for *P. aeruginosa* treated with NP^M123/Fc^+NIR‐II in relative to Mock (**Figure** **4**). First, the principal component analysis (PCA) indicated the datasets of these two groups were lasted at two different clusters, indicating the existence of obvious parallelism within each group but high‐level differences between the two groups (Figure 4a). Second, a total of 116 differentially regulated metabolites (DRMs) were identified for NP^M123/Fc^+NIR‐II compared Mock (Figure S18, Supporting Information), and they could be divided into 62 up‐regulated metabolites and 54 down‐regulated ones (Figure 4b,c). Third, as shown by Kyoto encyclopedia of genes and genomes (KEGG) enrichment analysis, there were the four enriched up‐regulated metabolic pathways (i.e., biosynthesis of secondary metabolites, arginine and proline metabolism, aminobenzoate degradation, and nicotinate and nicotinamide metabolism), and also the nine enriched down‐regulated metabolic pathways especially including pyrimidine metabolism, *β*‐alanine metabolism, alanine, aspartate and glutamate metabolism, glutathione metabolism, and purine metabolism (Figure 4d). Fourth, the major DRMs and related pathways were mapped, aiming to interpret bacterial metabolic dysregulation (Figure 4e): i) purine metabolism‐related GTP, dGMP, and deoxyguanosine were down‐regulated, thereby obstructing DNA synthesis and causing DNA damage^[^ ^24^ ^]^; ii) pyrimidine metabolism CMP and cytosine were down‐regulated, hence hampering RNA synthesis^[^ ^25^ ^]^; iii) riboflavin metabolism‐related riboflavin were up‐regulated, denoting over‐production of ROS^[^ ^26^ ^]^; iv) glutathione metabolism‐related putrescine and NADP^+^ were up‐regulated, consequently leading to enhancement of oxidative stress; and v) carbon metabolism‐related succinate and O‐acetyl‐L‐serine were down‐regulated, therefore resulting in disturbance of energy metabolism.^[^ ^27^, ^28^ ^]^ In addition, as revealed by Receiver Operating Characteristic (ROC) curve analysis (Figure S19, Supporting Information), putrescine gave an AUC (area under the ROC curve) value of 0.990 (> the cutoff 0.9^[^ ^29^ ^]^); therefore, the up‐regulation of putrescine (as a biomarker) denoted the pathological state in *P. aeruginosa* after treatment. Taken together, NP^M123/Fc^+NIR‐II induced inhibited nucleic acid synthesis, disordered energy metabolism, enhanced oxidative stress, and elevated DNA damage in bacteria.

### 2.5 Therapeutic Efficacy Against Infected Wounds in Mice

**Figure 5:**
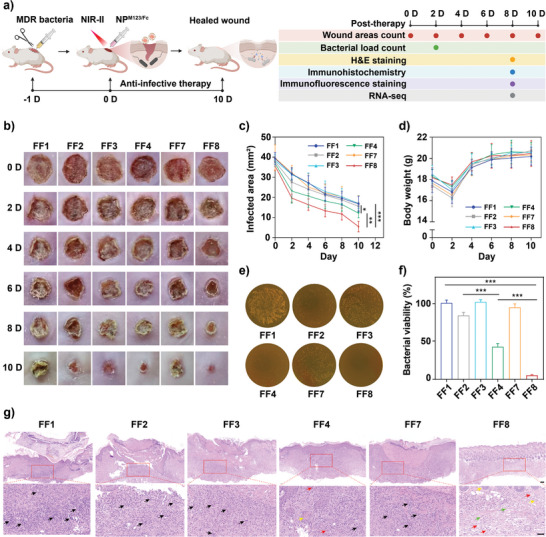
Therapeutic efficacy against *P. aeruginosa*‐infected wounds. FF1: Mock, FF2: NIR‐II, FF3: NP^M123^, FF4: NP^M123^+NIR‐II, FF7: NP^M123/Fc^, FF8: NP^M123/Fc^+NIR‐II. NIR‐II represents 1064 nm laser irradiation (1.0 W cm^−2^, 3.5 min). a) Schematic illustration of the orderly program of infected wound establishment and anti‐infective therapy, as well as the time points for different tests post therapy. b) Representative photos of infected wounds post therapy. Statistical analysis of c) wound areas and d) body weights post therapy. Data are presented as mean ± SD (*n* = 5). e) Representative photos of plate count agars for infected wounds on day 10 post‐therapy, and f) corresponding statistical analysis of bacterial load counts. Data are presented as mean ± SD (*n* = 6). g) Representative photos of H&E staining of infected wounds (*n* = 3) on day 10 post‐therapy. Scale bar is 100 µm. Black arrows denote inflammatory cells such as neutrophils, macrophages, and lymphocytes. Green arrows represent hair follicles. Yellow arrows show fibroblasts. Red arrows stand for new blood vessels. ns: *p* ≥ 0.05, *: *p* < 0.05, **: *p* < 0.01, ***: *p* < 0.001.

Endogenous H_2_O_2_ at infection sites would trigger the degradation of nanoparticles around infected bacteria, which would be along with the abovementioned synergistic antibacterial action under 1064 nm laser irradiation. Given this, in vivo therapeutic efficacy against infected wounds in mice was evaluated with an orderly program^[^ ^23^ ^]^ of wound fabrication, bacterial inoculation, infection establishment, and anti‐infective therapy (**Figure** **5**a). The infections involved two clinical MDR isolates: *S. aureus* USA300‐FPR3757^[^ ^30^ ^]^ and *P. aeruginosa* F291007.^[^ ^31^ ^]^ USA300‐FPR3757 belonged to the *S. aureus* USA300 clone, which had a highly virulent phenotype and represented a major source of community‐acquired *S. aureus* infections in the USA, Canada, and Europe.^[^ ^30^ ^]^ F291007 belonged to the high‐risk *P. aeruginosa* ST235 clone, which displayed a hypervirulent phenotype and caused worldwide nosocomial infections with poor clinical outcomes.^[^ ^32^ ^]^ Herein, a total of 5 therapeutic groups namely Mock, NIR‐II, NP^M123^, NP^M123^+NIR‐II, NP^M123/Fc^, NP^M123/Fc^+NIR‐II were employed. First, NP^M123/Fc^+NIR‐II and NP^M123^+NIR‐II gave almost the same tendency of temperature increase to about 48 °C within 3.5 min at *P. aeruginosa*‐infected wounds upon 1064 laser irradiation (Figure S20, Supporting Information). These local mild‐heat temperatures would readily cause thermal injury to bacterial cells but not surrounding tissues.^[^ ^10^ ^]^ Second, the wounds were photographed and their areas were counted for mice infected by *P. aeruginosa* (Figure 5b,c) or *S. aureus* (Figure S21a,b, Supporting Information) at sequential time points post‐therapy; compared to all the other therapeutic groups, NP^M123/Fc^+NIR‐II gave the most obvious decrease in wound areas and the smoothest scabs on day 10 post‐therapy. As for body weights, there was no statistically significant difference between all five groups (Figure 5d; Figure S21c, Supporting Information). Third, the bacterial loads at wounds on day 2 post‐therapy were counted for *P. aeruginosa‐* (Figure 5e,f) and *S. aureus‐*infected mice (Figure S21d,e, Supporting Information); NP^M123/Fc^+NIR‐II gave the largest inhibition rates (i.e., 94.88% and 90.98% for *P. aeruginosa* and *S. aureus*, respectively) in relative to all the other four groups. Fourth, as determined by hematoxylin and eosin (H&E) staining of wound tissues on day 10 post‐therapy for *P. aeruginosa‐* (Figure 5g) and *S. aureus‐*infected mice (Figure S21f, Supporting Information), NP^M123/Fc^+NIR‐II exhibited the best histopathologic outcome—abundant fibroblasts, regenerated blood vessels, and new hair follicles in granulation tissues; by contrast, there were abundant neutrophils, macrophages, and lymphocytes at wound tissues for all the other four groups, disclosing the persistence of infection‐induced inflammation at wounds. Taken together, NP^M123/Fc^+NIR‐II gave the best in vivo antibacterial and wound healing efficacy.

### 2.6 Host‐Response View of Anti‐Infective Mechanism In Vivo

**Figure 6:**
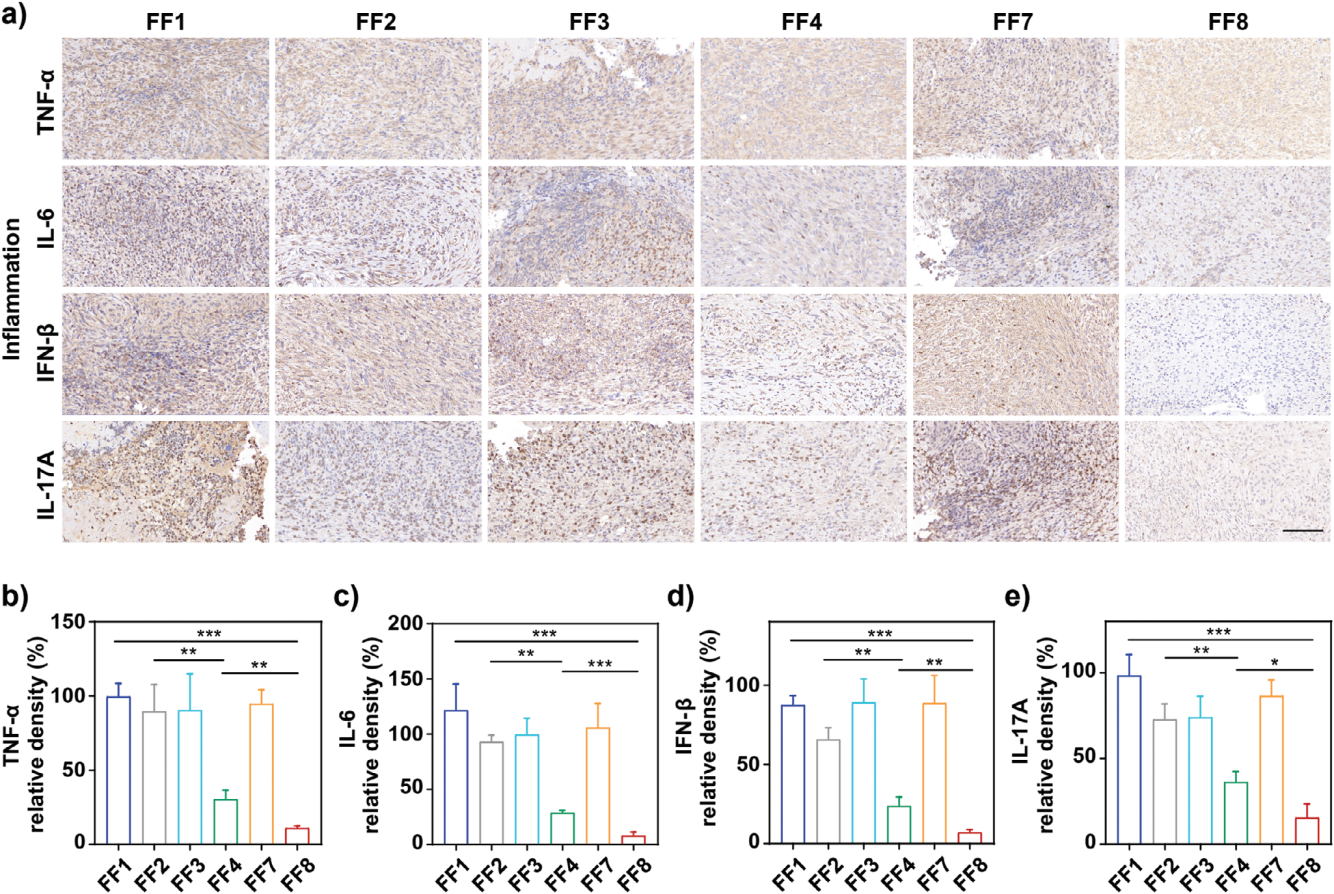
Immunohistochemistry assay of major proinflammatory cytokines in mice on day 8 post‐therapy. FF1: Mock, FF2: NIR‐II, FF3: NP^M123^, FF4: NP^M123^+NIR‐II, FF7: NP^M123/Fc^, FF8: NP^M123/Fc^+NIR‐II. NIR‐II represents 1064 nm laser irradiation (1.0 W cm^−2^, 3.5 min). (a) Representative immunohistochemical staining photos for TNF‐*α*, IL‐6, IFN‐*β*, and IL‐17A. Yellow brown represents positive detection. Scale bar = 100 µm. Corresponding statistical analysis for d) IL‐6, e) TNF‐*α*, f) IFN‐*β*, and g) IL‐17A. Data are expressed as mean ± SD (*n* = 3). ns: *p* ≥ 0.05, *: *p* < 0.05, **: *p* < 0.01, ***: *p* < 0.001.

**Figure 7:**
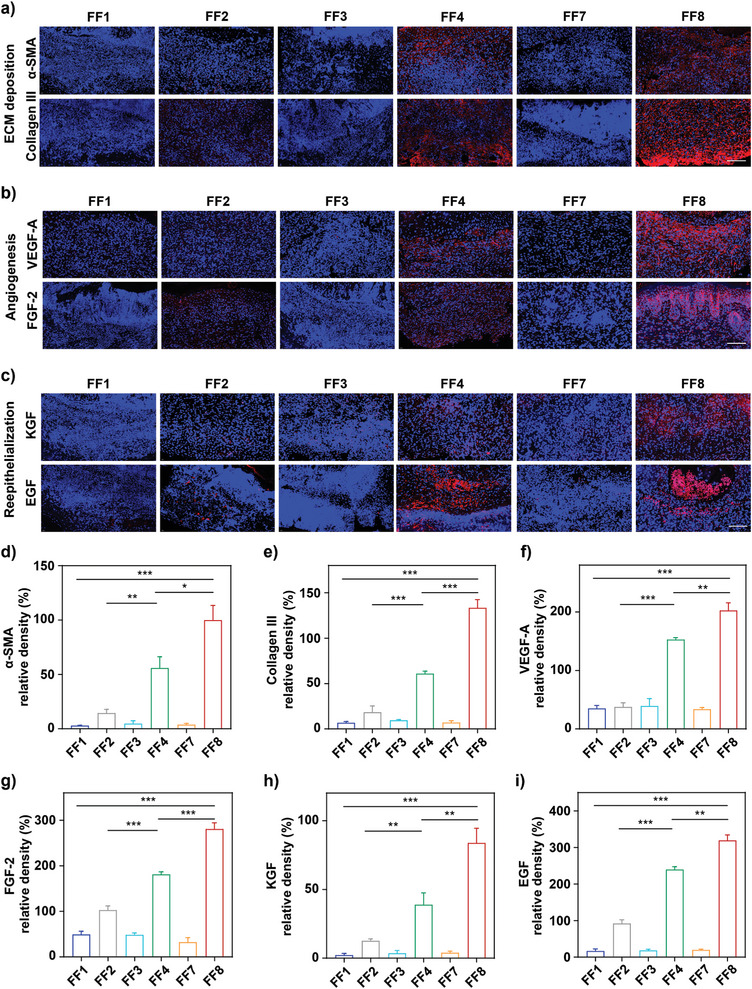
Immunofluorescence staining assay of major wound‐healing protein factors in mice on day 8 post‐therapy. FF1: Mock, FF2: NIR‐II, FF3: NP^M123^, FF4: NP^M123^+NIR‐II, FF7: NP^M123/Fc^, FF8: NP^M123/Fc^+NIR‐II. NIR‐II represents 1064 nm laser irradiation (1.0 W cm^−2^, 3.5 min). Representative immunofluorescence staining photos for a) *α*‐SMA and Collagen III, b) VEGF‐A and FGF‐2, and c) KGF and EGF. Red represents positive detection. Scale bar = 100 µm. Corresponding statistical analysis for d) *α*‐SMA, e) Collagen III, g) VEGF‐A, f) FGF‐2, h) KGF, and i) EGF. Data are expressed as mean ± SD (*n* = 3). ns: *p* ≥ 0.05, *: *p* < 0.05, **: *p* < 0.01, ***: *p* < 0.001.

To determine the expression of the four major proinflammatory cytokines including TNF‐*α*, IL‐6, IFN‐*β*, and IL‐17A,^[^ ^33^, ^34^ ^]^ immunohistochemistry was conducted with *P. aeruginosa*‐infected wound tissues on day 8 post‐therapy by Mock, NIR‐II, NP^M123^, NP^M123^+NIR‐II, NP^M123/Fc^, and NP^M123/Fc^+NIR‐II, and the results showed that the NP^M123/Fc^+NIR‐II resulted in the lowest production of each of these cytokines (**Figure** **6**), indicating that NP^M123/Fc^+NIR‐II enabled the best anti‐inflammatory effect. To measure the expression of the six major wound‐healing protein factors including *α*‐SMA and collagen III (extracellular matrix deposition), VEGF‐A and FGF‐2 (angiogenesis), and KGF and EGF (re‐epithelialization),^[^ ^35^, ^36^ ^]^ immunofluorescence staining was carried out with *P. aeruginosa*‐infected wound tissues on day 8 post‐therapy by the above six therapeutic groups, and the results indicated that NP^M123/Fc^+NIR‐II displayed the highest production of each of these six wound‐healing factors (**Figure** **7**), denoting that NP^M123/Fc^+NIR‐II brought the fastest wound‐healing process. Taken together, NP^M123/Fc^+NIR‐II exhibited the best efficacy to inhibit inflammatory reaction and meanwhile to promote wound healing.

**Figure 8:**
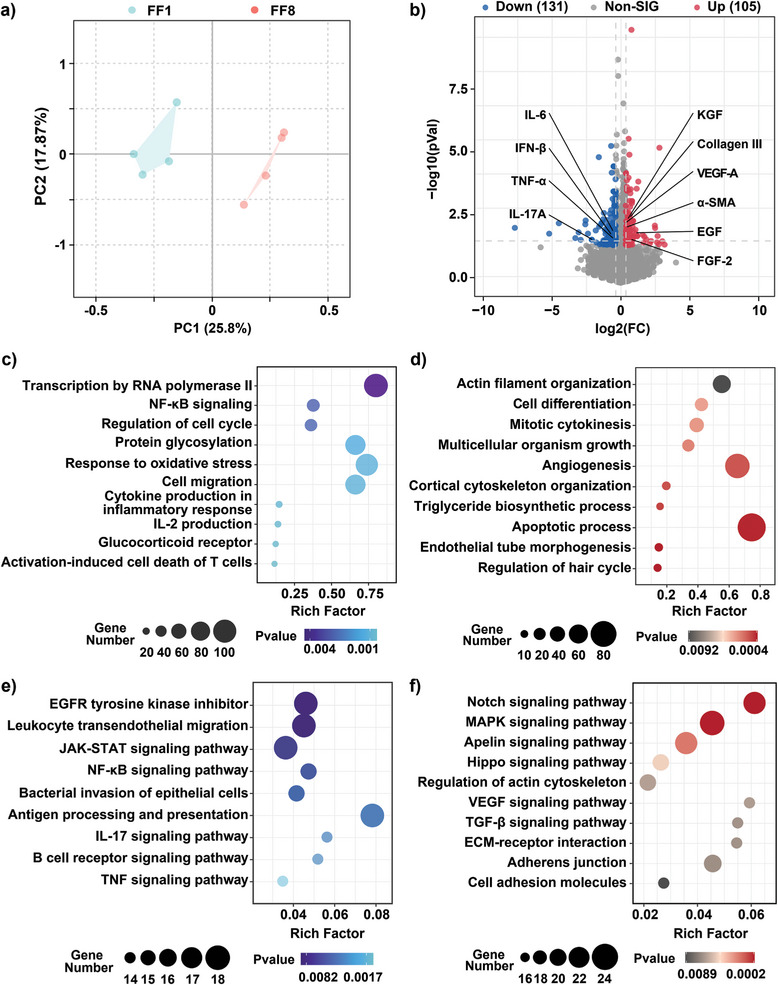
RNA‐seq assay of *P. aeruginosa*‐infected wounds on day 8 post‐therapy. a) PCA plot of clustered datasets between the two therapeutic groups NP^M123/Fc^+NIR‐II (FF8) and Mock (FF1). NIR‐II represents 1064 nm laser irradiation (1.0 W cm^−2^, 3.5 min). b) Volcano plot of DGEs for NP^M123/Fc^+NIR‐II compared to Mock. Up: Up‐regulated genes. Down: Down‐regulated genes. GO pathway enrichment analysis was performed for c) down‐regulated and d) up‐regulated genes, and KEGG pathway enrichment analysis was conducted for e) down‐regulated and f) up‐regulated genes. Experiments were performed with four independent biological replicates.

To characterize the global host response after anti‐infective therapy, RNA‐seq‐determined transcriptomes of *P. aeruginosa*‐infected wound tissues on day 8 post‐therapy was compared between the two therapeutic groups NP^M123/Fc^+NIR‐II and Mock (**Figure** **8**). First, the PCA plot (Figure 8a) showed the far distance between the clustered datasets of these two groups, indicating the existence of a high‐level of data reproducibility within each group as well as a high‐degree of data differentiation between these two groups. Second, a total of 236 differentially expressed genes (DEGs) were identified for NP^M123/Fc^+NIR‐II in relative to Mock, and they were composed of 131 down‐regulated genes that included those encoding the above four proinflammatory cytokines, and additionally 105 up‐regulated genes that included those encoding the above six wound‐healing factors (Figure 8b). Third, as determined by gene ontology (GO) pathway enrichment analysis of these DEGs, there were the five enriched down‐regulated pathways including NF‐κB signaling, IL‐2 production, regulation of cytokine production involved in inflammatory response, negative regulation of activation‐induced cell death of T cells, and negative regulation of glucocorticoid receptor signaling pathway (Figure 8c), and meanwhile the seven enriched up‐regulated pathways including positive regulation of apoptotic process, angiogenesis, actin filament organization, positive regulation of cell differentiation, cortical cytoskeleton organization, endothelial tube morphogenesis, and regulation of hair cycle (Figure 8d). Fourth, KEGG enrichment analysis gave similar results—there were the seven enriched down‐regulated pathways including the TNF signaling pathway, NF‐κB signaling pathway, IL‐17 signaling pathway, JAK‐STAT signaling pathway, antigen processing and presentation, B cell receptor signaling pathway, leukocyte transendothelial migration (Figure 8e), and simultaneously the nine enriched up‐regulated pathways including ECM‐receptor interaction, regulation of actin cytoskeleton, adherens junction, cell adhesion molecules, VEGF signaling pathway, TGF‐*β* signaling pathway, Notch signaling pathway, Apelin signaling pathway, and Hippo signaling pathway (Figure 8f). Notably, all the above enriched down‐regulated pathways were involved in the resolution of excessive inflammation, while all these enriched up‐regulated pathways contributed to wound healing, giving a global view of the coordinated process of clearance of pathogenic stimuli along with restoration of tissue integrity and function. Fifth, the heatmap in Figure S22 (Supporting Information) presented not only the ten factors simultaneously revealed by the traditional immunoassay and the RNA‐seq assay, but also the 12 selected down‐regulated proinflammatory factors and the 19 selected up‐regulated wound‐healing factors additionally disclosed by RNA‐seq, all of which would be the major contributors to the highly favored therapeutic effect of NP^M123/Fc^+NIR‐II. Taken together, the therapeutic effect with respective to host response was mainly composed of inhibition of inflammatory reaction and meanwhile promotion of wound healing.

### 2.7 Biosafety at Cellular and Animal Levels

First, the embryonic mouse normal fibroblast cell line NIH‐3T3 and the human normal ovarian epithelial cell line IOSE‐80 were used to test the potential cytotoxicity of NP^M123/Fc^ by using Cell Counting Kit‐8 (CCK8), and each cell line gave a cell viability >80%^[^ ^14^, ^23^, ^31^ ^]^ after co‐incubation with 100 µg mL^−1^ NP^M123/Fc^ for 12 h (Figure S23, Supporting Information). The additional hemolysis assay with mouse red blood cells (RBCs) revealed that 100 µg mL^−1^ NP^M123/Fc^ presented a hemolytic rate <5% (Figure S24, Supporting Information).^[^ ^14^, ^23^, ^31^ ^]^ Second, mice with infection‐free wounds were treated with Mock, NIR‐II, NP^M123^, NP^M123^+NIR‐II, NP^M123/Fc^, NP^M123/Fc^+NIR‐II using the administration route and dosage same as anti‐infective therapy. H&E staining on day 8 post‐treatment showed no inflammatory or pathological lesions in heart, liver, spleen, lung, and kidney (Figure S25, Supporting Information). After treatment with 1 mg kg^−1^ lipopolysaccharide (LPS), obvious infiltration of neutrophils, macrophages, and lymphocytes were observed at wounds (positive control) but not in all the five organs (Figure S25, Supporting Information). Biochemical blood test at the same time point exhibited no abnormal changes in the two major hepatic function indexes namely alanine aminotransferase (ALT), aspartate aminotransferase (AST) and the two major renal function indexes namely blood urea nitrogen (BUN) and creatinine (CREA) (Figure S26, Supporting Information). Third, for *S. aureus*‐ or *P. aeruginosa*‐infected wounds treated with the above six groups, and H&E staining (Figure S27, Supporting Information) and biochemical blood test (Figure S28, Supporting Information) on day 8 post‐therapy gave the similar biosafety results; it should be noted that, after wounds were infected with bacteria, there was significant inflammatory reaction at infected wounds, but no obvious pathological injury was found in the heart, liver, spleen, lung, and kidney whether the therapeutic measures were applied or not. Taken together, NP^M123/Fc^ had highly favorable biosafety at cellular and animal levels.

## 3 Conclusion

The present work developed a novel polymer nanomaterial NP^M123/Fc^ for treatment of acute wound infections. We used two distinct polymers, namely the photothermal agent M123‐containing P^M123^ and the Fc‐containing P^Fc^ both containing ROS‐sensitive moieties, to be assemble into NP^M123/Fc^. Fc was built in a polymerized manner at the center locations of NP^M123/Fc^, and thus Fc introduced was highly controllable at high consternations. In addition, Fc as a CDT agent has been showed higher levels of biocompatibility relative to inorganic CDT metals.^[^ ^16^, ^17^, ^18^ ^]^ As expected, M123 in NP^M123/Fc^ endowed the efficient and stable photothermal effect upon 1064 nm laser irradiation and, Via Fenton‐like reaction, Fc in NP^M123/Fc^ enabled the chemodynamic effect to produce more toxic ∙OH from endogenous H_2_O_2_ abundant at infection sites. These two distinct antibacterial contributors PTT and CDT worked in a strong synergistic manner. NP^M123/Fc^+NIR‐II gave >90% in vitro inhibition rates against MDR ESKAPE pathogens. The metabolomics analysis disclosed NP^M123/Fc^+NIR‐II in vitro induced bacterial metabolic dysregulation especially including inhibited nucleic acid synthesis, disordered energy metabolism, enhanced oxidative stress, and elevated DNA damage. NP^M123/Fc^+NIR‐II possessed >90% bacteriostatic rates in a mouse model of cutaneous wounds infected by *P. aeruginosa* and *S. aureus*, resulting in almost full recovery of infected wounds. The systematic immunohistochemistry, immunofluorescence staining, and transcriptomics assays with respective to host response disclosed that the therapeutic effect was mainly dependent on inhibition of inflammatory reaction and meanwhile promotion of wound healing at wound sites. The ROS‐sensitive thioketal bonds in NP^M123/Fc^ made it degradable upon ROS. Endogenous H_2_O_2_ at infection sites would trigger the degradation of NP^M123/Fc^ located around infected bacteria, and ∙OH generated by CDT would further promote this biodegradation. On the one hand, the rapid degradation of NP^M123/Fc^ endowed highly favorable biosafety as observed at both cellular and animal levels. On another hand, this rapid degradation would be along with the quick release of M123 (PTT) and Fc (CDT) for subsequent rapid bactericidal actions. NP^M123/Fc^ with a unique synergistic PTT and CDT strategy represented a biocompatible and broad‐spectrum nanoplatforms for antibiotics‐alternative treatment of acute superficial infections.

## Conflict of Interest

The authors declare no conflict of interest.

## Supporting information

Supporting Information

## Data Availability

The data that support the findings of this study are available from the corresponding author upon reasonable request.
