# Supplementary material for: Treatment of Acute Wound Infections by Degradable Polymer Nanoparticle with a Synergistic Photothermal and Chemodynamic Strategy
Source: Adv Sci (Weinh). 2024 Feb 26;11(17):2309624. doi: 10.1002/advs.202309624 (PMC11077640; doi:10.1002/advs.202309624)
Supplement: Supplementary file 1 — Supporting Information [file ADVS-11-2309624-s001.pdf]

ADVANCED SCIENCE
Open Access

Supporting Information

for *Adv. Sci.*, DOI 10.1002/advs.202309624

Treatment of Acute Wound Infections by Degradable Polymer Nanoparticle with a Synergistic Photothermal and Chemodynamic Strategy

Fangzhou Chen, Lin Liu, Dongsheng Tang, Hanchen Zhang, Nier Wu, Lin Wang, Hongbo Li, Haihua Xiao* and Dongsheng Zhou**

Supporting Information

Treatment of Acute Wound Infections by Degradable Polymer Nanoparticle with a Synergistic Photothermal and Chemodynamic Strategy

Fangzhou Chen#, Lin Liu#, Dongsheng Tang, Hanchen Zhang, Nier Wu, Lin Wang, Hongbo Li, Haihua Xiao*, Dongsheng Zhou**

Table of Contents

Experimental Section.

Scheme S1. Schematic illustration of preparation of NP^{M123} and NP^{Fc}.

Figure S1. ^1H NMR spectrums of P^{M123}.

Figure S2. ^1H NMR spectrums of P^{Fc}.

Figure S3. TEM images of NP^{M123} and NP^{Fc}.

Figure S4. SEM images of NP^{M123}, NP^{Fc}, and $NP^{M123/Fc}$.

Figure S5. Thermal imaging for $NP^{M123/Fc}$ under 1064 nm laser irradiation (1.0 W cm^{-2}, 5 min).

Figure S6. Time–concentration–temperature curves of NP^{M123} under 1064 nm laser irradiation (1.0 W cm^{-2}, 5 min).

Figure S7. Time-dependent heating and cooling curve of 100 μg mL^{-1} $NP^{M123/Fc}$ under 1064 nm laser irradiation (1.0 W cm^{-2}, 11 min). The η value was calculated, through linear regression, from this heating and cooling curve.

Figure S8. Time–temperature curve of 4 cycles of heating and cooling of NP^{M123} under 1064 nm laser irradiation (1.0 W cm^{-2}, 5 min).

Figure S9. Absorption curves of DPBF as a selective trapping agent to detect ROS in the reaction mixture of $NP^{M123}+H_2O_2$, $NP^{Fc}+H_2O_2$, or $NP^{M123/Fc}+H_2O_2$ after different incubation times.

Figure S10. Trends of UV absorption at 410 nm as indicated by the above DPBF-based detection results.

Figure S11. a) Absorption curves of MB as a selective trapping agent to detect •OH in the reaction mixture of $NP^{Fc}+H_2O_2$ after different incubation times. b) Trends of UV absorption at 410 nm as indicated by the above MB-based detection results.

Figure S12. Fc release kinetics of $NP^{M123/Fc}$ *in vitro* was determined by ICP-MS.

Figure S13. XPS spectra of P^{Fc}.
Figure S14. TEM images for NP^{M123}, NP^{Fc} and $NP^{M123/Fc}$ in the presence of H_2O_2.
Figure S15. SEM images for NP^{M123}, NP^{Fc} and $NP^{M123/Fc}$ in the presence of H_2O_2.
Figure S16. DLS measurement of diameter sizes and PDIs of a) NP^{M123} and b) NP^{Fc} in the presence or absence of H_2O_2. c) Average Zeta potentials of NP^{M123} and NP^{Fc} in the presence or absence of H_2O_2.
Figure S17. TEM images of *P. aeruginosa* and *S. aureus* post treatment.
Figure S18. Classification of DRMs.
Figure S19. ROC curve for DRMs.
Figure S20. Time-dependent temperature increase at *P. aeruginosa*-infected wounds.
Figure S21. Therapeutic efficacy against *S. aureus*-infected wounds.
Figure S22. Heatmap of selected DRGs involved in proinflammation and wound healing genes.
Figure S23. Viability of NIH-3T3 and IOSE-80 cells incubated with $NP^{M123/Fc}$ at different concentrations for 24 h.
Figure S24. Hemolysis rates of RBCs incubated with different concentrations of $NP^{M123/Fc}$.
Figure S25. H&E staining of major organs in infection-free mice (n = 3) on day 8 post-treatment.
Figure S26. Detection of serum biochemical indicators including a) ALT (Reference range: 10.06-96.47 U L^{-1}), b) AST (Reference range: 36.31-235.48 U L^{-1}), c) BUN (Reference range: 10.81-34.74 mg dL^{-1}), and d) CREA (Reference range: 10.91-85.09 µmol L^{-1}) in infection-free mice (n = 3) on day 8 post-treatment.
Figure S27. H&E staining of major organs in a) *P. aeruginosa*- and b) *S. aureus*-infected mice (n = 3) on day 10 post-therapy.
Figure S28. Detection of serum biochemical indicators a) ALT, b) AST, c) BUN, and d) CREA of *P. aeruginosa*-infected and e) ALT, b) AST, c) BUN, and d) CREA in *S. aureus*-infected mice (n = 3) on day 10 post-therapy.

Experimental Section

Materials. 5-Bromo-2-thiophenecarboxylic acid and tris (dibenzylideneacetone) dipalladium (dba_3pd_2) (CAS: 51364-51-3) were purchased from Aladdin (Shanghai, China). 1-(3-Dimethylaminopropyl)-3-ethylcarbodiimide was purchased from Sigma-Aldrich (St. Louis, MO, USA). Tri(o-tolyl) phosphine ($P(o\text{-}tol)_3$) (CAS: 6163-58-2) and Cy7.5 were purchased from Energy Chemical (Shanghai, China). 2,5-Bis(2-ethylhexyl)-3,6-bis(5-(trimethylstannyl)thiophen-2-yl)pyrrolo[3,4-c]pyrrole-1,4(2H,5H)-dione (DPP26-2Sn), 2,6-

Dibromo-4,4-bis(6-bromohexyl)-4H-cyclopenta[2,1-b:3,4-b']dithiophene (DTCC6Br4), and 4,9-dibromo-6,7-bis(4-hexylphenyl)-[1,2,5]thiadiazolo[3,4-g]quinoxaline(DPTQ6-2Br) were purchased from Alfa (Zhengzhou, China). DSPE-mPEG$_{2000}$ were purchased from Sigma-Aldrich (St. Louis, MO, USA). Methanol, toluene, acetonitrile, glutaraldehyde, tetrahydrofuran, and ethanol were purchased from Sinopharm Chemical Reagent Co., Ltd (Shanghai, China). Brain Heart Infusion (BHI) were purchased from BD Biosciences (San Jose, CA, USA). CCK8-kit were purchased from MedChemExpress (Shanghai, China). SYTO-9/PI were purchased from Roche Applied Science (Basel, Switzerland). Dulbecco's modified Eagle medium (DMEM), fetal bovine serum (FBS), PBS, and trypsin-EDTA solution were purchased from Corning Inc. (New York, USA). All chemicals were used as received without further purification.

Synthesis of M4. M4 was synthesized as descried previously.[1] 2,2′-(Propane-2,2-diylbis (sulfanediyl) bis (ethan-1-ol) (1.98 g, 10.10 mmol), 5-bromothiophene-2-carboxylic acid (5.22 g, 25.25 mmol), 1-ethyl-3-(3-dimethylaminopropyl) carbodiimide hydrochloride (4.84 g, 25.25 mmol), and 4-dimethylaminopyridine (3.08 g, 25.25 mmol) were dissolved in 100 mL N, N-Dimethylformamide (DMF) and incubated at room temperature for 24 h. The reaction mixture was poured into 500 mL water, then extracted with 100 mL ethyl acetate for three times, and separated by column chromatography to obtain a white solid M4 with a yield of 66%.

Preparation of P^{M123}. P^{M123} was synthesized as descried previously.[1] M1 (80 mg, 0.094 mmol), M2 (21.80 mg, 0.033 mmol), M3 (31.35 mg, 0.047 mmol), M4 (8.10 mg, 0.014 mmol), dba_3Pd_2 (1.79 mg, 0.0019 mmol) were dissolved in 5 mL of degassed toluene, protected by nitrogen gas, and incubated at 120 °C for 3 h, and then the reaction solution was dropped into 500 mL of anhydrous methanol, stood for 30 min, filtered to obtain dark green precipitation, and finally dried to obtain P^{M123}.

Preparation of P^{Fc}. P^{Fc} was synthesized as descried previously.[2] L-lysine diisocyanate (260 mg, 1.15 mmol) was quickly added to an anhydrous DMF solution (5 mL) of 1,1'-ferrocenedimethanol (47.6 mg, 0.2 mmol) and ROS-sensitive linker (2,2'-(propane-2,2-diylbis(sulfanediyl))bis(ehan-1-ol)) (157 mg, 0.8 mmol). After magnetic stirring for another 12 h at room temperature, mPEG$_{5000}$-OH (1 g, 0.2 mmol) was added to the reaction mixture. After magnetic stirring for another 24 h at 50 ℃, the mixture was added into 10 mL of deionized

water under sonication, followed by dialysis in a dialysis bag (MWCO: 8000-14000 Da). After 72 h, the solution was freeze-dried under reduced pressure to give P^{Fc} (800 mg) powder.

Preparation of NP^{M123}, NP^{Fc} and $NP^{M123/Fc}$. DSPE-mPEG$_{2000}$ (10.0 mg) and P^{M123} (1.0 mg) were completely dissolved with 1 mL of tetrahydrofuran (THF) by bath sonication. DSPE-mPEG$_{2000}$ (10.0 mg) and P^{Fc} (1.0 mg) were completely dissolved with 1 mL of THF by bath sonication. DSPE-mPEG$_{2000}$ (10.0 mg), P^{M123} (1.0 mg), and P^{Fc} (2.0 mg) were completely dissolved with 1 mL of THF by bath sonication. The mixture was added quickly into deionized water (50.0 mL) under continuous sonication, and THF in the solution was then removed by dialysis with a dialysis bag (molecular weight cutoff, 3500 Da) for 24 h, obtaining the NP^{M123}, NP^{Fc}, and $NP^{M123/Fc}$ solutions, respectively.

Characterizations. 5 mg P^{M123} or P^{Fc} was dissolved in deuterated THF, and ^{1}H NMR spectra was subsequently measured by a 400 MHz NMR spectrometer (Bruker) at room temperature. 20 μg mL^{-1} NP^{M123}, NP^{Fc}, or $NP^{M123/Fc}$ aqueous solution was prepared and then TEM and SEM images were observed by Hitachi HT-7700 TEM (Japan) and JEM-ARM200F (Japan) respectively, while STEM and EDXS images were recorded by JEOL JEM-2100F STEM (USA). 20 μg mL^{-1} $NP^{M123/Fc}$ aqueous solution was prepared and then UV–Vis–NIR spectra in wavelength range from f 350 nm to 1250 nm was recorded using a spectrophotometer (TU-1901).

***In Vitro* Photothermal Measurement.** A series of NP^{M123} or $NP^{M123/Fc}$ at different concentrations (200, 100, 50, 25, and 0 μg mL^{-1}, respectively) were prepared and irradiated with 1064 nm laser (1.0 W cm^{-2}, 5 min), and then the temperature changes were recorded by infrared thermal imaging camera (FLIR A6501, USA). For determination of photothermal conversion efficiency, NP^{M123} or $NP^{M123/Fc}$ was continuously irradiated by 1064 nm laser (1.0 W cm^{-2}, 11 min) until the temperatures were increased to a highest steady state, and then the laser was turned off until the temperatures were cooled to a lowest steady state; after that, the aqueous dispersion was cooled at room temperature and the photothermal conversion efficiency (η) of $NP^{M123/Fc}$ was calculated by the following equation:[3]

$$\eta = \frac{Q_{sample} - Q_{water}}{I\,(1-10^{-A_\lambda})} = \frac{h*A\,[T_{Max(sample)} - T_{surr(sample)}] - h*A\,[T_{Max(water)} - T_{surr(water)}]}{I\,(1-10^{-A_\lambda})}$$

$$h*A = \frac{m_{water} * C_{water}}{\tau_s} \qquad t = -\tau_s \ln(\theta)$$

Where *h* was the heat-transfer coefficient, T_{max} was the equilibrium temperature, T_{surr} was the ambient temperature, *I* was the density of laser power, A_{λ} was the absorbance of $NP^{M123/Fc}$ at 1064 nm, and τ_s was the time constant that was calculated by the fitting curve of time versus $-\ln\theta$ obtained from Figure 1e. For photothermal stability measurement, NP^{M123} or $NP^{M123/Fc}$ (100 µg mL^{-1}) was exposed to 1064 nm laser irradiation (1.0 W cm^{-2}, 5 min) for 4 ON/OFF cycles, and the temperature variation of the solution was recorded by infrared thermal imaging camera (FLIR A6501, USA).

***In Vitro* Chemodynamic Study.** DPBF assay was used to measure the ROS generation efficacy of NP^{M123}, NP^{Fc} and $NP^{M123/Fc}$, and all the experiments were carried out at room temperature. 100 µg mL^{-1} NP^{M123}, NP^{Fc} or $NP^{M123/Fc}$ was mixed with 1 mM H_2O_2 and 2 mM DPBF, and tested at every half minute for a total duration time of 3.5 min under the NIR-II laser (1064 nm, 1.0 W cm^{-2}). UV–vis–NIR spectrophotometer was used to record the degradation curve of DPBF. The absorbance changes of DPBF at 410 nm were used to quantify the decomposition rate. MB assays were used to measure the •OH production efficacy of NP^{M123}, NP^{Fc} or $NP^{M123/Fc}$, and all the experiments were carried out at room temperature; 10 µg mL^{-1} of MB, 1 mM H_2O_2, and 100 µg mL^{-1} samples were incubated for different times (0 to 3.5 min, tested every half minute) under the NIR-II laser (1064 nm, 1.0 W cm^{-2}), and UV–vis–NIR spectrophotometer was used to record the degradation curve of MB. The absorbance changes of MB at 664 nm were used to quantify the decomposition rate. The Fc release kinetics of $NP^{M123/Fc}$ *in vitro* was measured by the dialysis method; a sealed dialysis bag (molecular retention of 3500) containing 5 mL of $NP^{M123/Fc}$ (100 µM Fc) was immersed in 200 mL of PBS in the presence or absence of 10 mM H_2O_2, followed by shaking at 100 rpm at room temperature. At various time points (0, 1, 2, 4, 6, 8, 10, 12, 24, 48 h), 1.5 mL of sample solution was taken from the dialysate and measured by ICP-MS to detect released Fc. XPS examination of P^{Fc} (1 mg mL^{-1}, 1 mL) was challenged with H_2O_2 (1 mM, 1 mL) at 37 °C for 24 h, and the solution was freeze-dried under reduced pressure and the resulting lyophilized samples were then tested by XPS.

Disintegration of Nanoparticles Triggered by H_2O_2. 100 µg mL^{-1} NP^{M123}, NP^{Fc} or $NP^{M123/Fc}$ was mixed with 1 mM H_2O_2 at 37 °C for 3.5 min. The solution was diluted to 20 µg mL^{-1} and then TEM and SEM images were recorded. Meanwhile, hydrodynamic diameters and zeta potentials were measured on Malvern Zetasizer Nano ZS90 (Malvern Instruments, UK).

Bacterial Strains and Cultivation. Clinical MDR ESKAPE isolates used in this study included *Ec. faecium* HJP554,[4] *S. aureus* USA300-R (USA300-FPR3757),[4-5] *K. pneumoniae* ATCC BAA-2146,[6] *A. baumannii* LAC-4,[4, 7] *P. aeruginosa* F291007,[4] and *Eb. hormaechei* ATCC BAA-2082 (https://www.atcc.org/products/baa-2082). All these 6 isolates were used for *in vitro* antibacterial assays, while *S. aureus* USA300-R and *P. aeruginosa* F291007 were employed as representatives for *in vivo* anti-infective assays. For bacterial cultivation, bacterial stock solution (30 μL) was inoculated in Brain-Heart Infusion (BHI) broth (3 mL) and incubated in a shaker at 37 °C for 15 h (200 rpm), which was the first-generation culture at the stationary stage. Subsequently, the mixture (30 μL) was inoculated in BHI broth (3 mL) and incubated in a shaker at 37 °C for 3 h (200 rpm), which was the second-generation culture at the middle logarithmic phage. Then, this culture was inoculated in BHI broth (3 mL) at a ratio of 1:100 and incubated in a shaker at 37 °C for 3 h (200 rpm), which was the third-generation culture at the middle logarithmic phage. Finally, the OD_{600} value of this culture was adjusted to 0.2 with a concentration of about 10^7 CFU mL^{-1} for subsequent experiments. For bacterial cultivation using BHI broth (see above) or BHI plate (see below), the following antibiotics were added: methicillin (5 μg mL^{-1}) for *S. aureus*, vancomycin (6 μg mL^{-1}) for *Ec. faecium,* ceftazidime (20 μg mL^{-1}) for *A. baumannii*, and meropenem (4 μg mL^{-1}) for *P. aeruginosa, K. pneumoniae*, and *Eb. hormaechei*.

***In vitro* Antibacterial Assay.** The *in vitro* antibacterial efficacy against ESKAPE bacteria was determined by counting the number of colony-forming units (CFUs). Experiments were divided into 8 treatment groups: Mock (FF1), NIR-II (FF2), NP^{M123} (FF3), NP^{M123}+NIR-II (FF4), NP^{Fc} (FF5), NP^{Fc}+NIR-II (FF6), $NP^{M123/Fc}$ (FF7), $NP^{M123/Fc}$+NIR-II (FF8) as employed as the dispersion solution, and the pure buffer was set as the FF1 treatment group. NIR-II represented 1064 nm laser irradiation (1.0 W cm^{-2}, 3.5 min). For each treatment group, bacteria (100 μL, 10^7 CFU mL^{-1}) were mixed with NP^{M123}, NP^{Fc}, or $NP^{M123/Fc}$ (100 μg mL^{-1}) in 1 mL of phosphate buffer saline (PBS, 0.1 M, pH = 7.4) with the addition of 1 mM H_2O_2. Subsequently, the mixture was diluted 100 times with PBS (0.1 M, pH = 7.4) and the diluted mixtures (each 100 μL) were plotted on BHI plates and cultured at 37 °C for 18 h to be used for photograph; meanwhile, the mixture was diluted 1000 times by PBS (0.1 M, pH = 7.4), and 10 μL of the diluted mixtures were plotted on BHI plates to be used for CFU counting. Finally, the number of CFUs was counted, followed by the calculation of bacterial viability using the following equation:

$$\text{Bacterial Viability \%} = \frac{\text{CFU}_{(\text{experimental group})}}{\text{CFU}_{(\text{control})}} \times 100\%$$

Where $CFU_{(control)}$ was the CFU value of the FF1 treatment group, and $CFU_{(experimental\ group)}$ was the CFU value of each of the FF2 to FF8 treatment groups.

Calculation of Q Value: Q value was calculated by the following equation:

$$Q_{A+B} = \frac{E_{A+B}}{E_A + E_B - E_A * E_B}$$

Where E_A and E_B were the *in vitro* antibacterial efficacy mediated by the photothermal effect of NP^{M123}+NIR-II (FF4) and that attributive to the chemodynamic effect of $NP^{M123/Fc}$ (FF7), respectively, and E_{A+B} was the overall *in vitro* antibacterial efficacy of $NP^{M123/Fc}$+NIR-II (FF8) combined with photo-therapy. The Q value reflected the synergistic effect between chemodynamic effect and photothermal effect: $Q > 1.15$ suggested strong synergism and $0.85 < Q < 1.15$ indicated an additive effect, while $Q < 0.85$ indicated an antagonism effect[8-10].

Live/Dead Fluorescent Staining. The viability of bacteria cells was qualitatively assessed through SYTO-9/PI staining. Bacteria (100 µL, 10^7 CFU mL^{-1}) were treated as described *in vitro* antibacterial assay until incubation in a shaker at 37°C for 30 min, and then the mixture was incubated with SYTO-9/PI for 20 min in the dark. The stained bacteria were observed with confocal laser scanning microscope (CLSM, ZEISS LSM 880).

Characterization of Bacterial Morphology. Bacteria (100 µL, 10^7 CFU mL^{-1}) were treated as described in *in vitro* antibacterial assay, and then the mixture was centrifuged (6000 rpm) for 5 min, washed twice with PBS (0.1M, pH = 7.4), and fixed by 4% paraformaldehyde for 2 h. Subsequently, the suspension was gradually dehydrated at different concentrations of ethanol (50%, 70%, 90%, and 100%, respectively) for 10 min, respectively, and then SEM or TEM (see above) was used to observe the changes in bacterial morphology and the bacterial adherence to nanoparticles.

Evaluation of DNA, Protein, K^+ and Na^+ Leakage. Bacteria (100 µL, 10^7 CFU mL^{-1}) were treated as described in *in vitro* antibacterial assay until incubation in a shaker at 37 °C for 30 min. For the detection of the levels of DNA, protein, K^+, and Na^+ released by bacteria, bacterial supernatant was collected by centrifugation (6000 rpm) for 5 min. OD_{260nm} value determined by Nano Drop UV–Vis spectrophotometer reflected the amount of released DNA. Protein level was determined by using BCA Protein Assay Kit (#23227, Thermo-Fisher, Grand Island, NY). In addition, K^+ and Na^+ levels were measured by ICP-MS.

DNA Damage Staining. Bacteria (100 µL, 10^7 CFU mL^{-1}) were treated as described in the above *in vitro* antibacterial assay, and then the mixture was incubated with DAPI for 20 min in the dark, and then stained bacteria were observed with CLSM (see above).

Bacterial Metabolomics Assay. *P. aeruginosa* (100 µL, 10^8 CFU mL^{-1}) was treated *in vitro* by the $NP^{M123/Fc}$+NIR-II group (giving a bacterial viability rate of about 30%) or by the Mock group, and then total metabolites were analyzed by liquid chromatography with tandem mass spectrometry (LC-MS/MS). Differentially regulated metabolites (DRMs) were identified as the metabolites with variable importance in the projection (VIP) value > 1, $|\log_2(\text{fold change})| > 1$, and student's t-test P-value < 0.05.

***In Vivo* Antibacterial and Wound Healing Evaluation.** BALB/c female mice (6~8 weeks, about 18 g) were purchased from Vital River Laboratories (Beijing, China). A wound approximately 7 mm in diameter was made on the dorsum of each mouse, and bacterial suspension (10 µL, 10^7 CFU mL^{-1}) was dropped into each wound. After the wounds were infected for 24 h, the mice were divided randomly into six therapeutic groups: Mock (FF1), NIR-II (FF2), NP^{M123} (FF3), NP^{M123}+NIR-II (FF4), $NP^{M123/Fc}$ (FF7), and $NP^{M123/Fc}$+NIR-II (FF8) as above. NIR represented laser irradiation (1064 nm, 1.0 W cm^{-2}, 3.5 min), which was used 1.5 cm away from wound so that the light could cover the entire wound. The changes in temperature at infected wounds post therapy were recorded every half minute for a total during time of 3.5 min by an infrared thermal imaging camera (see above). Photographs of wounds were taken on days 0, 2, 4, 6, 8, and 10 post therapy, and the sizes of wounds and the body weights of mice were measured simultaneously. The mice were sacrificed on day 10 post therapy and the primary organs including heart, liver, spleen, lung, kidney, and wound tissues were harvested, fixed in 4% paraformaldehyde overnight, and dehydrated, and then embedded in paraffin for pathological histology, immunohistochemistry, and immunofluorescence staining.

Histology, Immunohistochemistry, Immunofluorescence Staining, and RNA-seq. The organ or wound tissues of infected mice on day 8 post-therapy were sliced. The slices (4 µm in thickness) were gradually dewaxed in xylene for 15 min and washed with 75%, 85% and 95% ethanol, respectively, for 5 min.

For hematoxylin and eosin staining, the dewaxed slices were incubated with hematoxylin for 5 min and washed with water for three times, and gradually dehydrated with 75%, 85% and 95% ethanol, respectively, for 5 min, followed by incubation with eosin for 5 min. Furthermore, the slices were washed with ethanol for 5 min and three times, and then with xylene for 5 min and two times. The results were observed by a light microscope (Nikon Eclipse E100).

For immunohistochemistry, the dewaxed slices were incubated with the primary antibody against each of TNF-α (Servicebio, GB11188), IL-6 (Servicebio, GB11020), IFN-β (Servicebio, GB300051), and IL-17A (Servicebio, GB300036) overnight in a wet box at 4 °C. Afterward, the slices were washed with PBS (0.1 м, pH = 7.4) in a rocker device for three times, and incubated with the corresponding goat anti-rabbit secondary antibody labeled with horseradish peroxidase (HRP) at a room temperature for 45 min in dark condition. Then, the slices were washed with 75%, 85%, and 95% ethanol, respectively, for 5 min, and with xylene for 5 min. The results were observed by microscope (XSP-C204).

For immunofluorescence staining, the dewaxed slices were incubated with the primary antibody against each of α-SMA (Servicebio, GB13044), collagen III (Servicebio, GB13023-2), VEGF-A (Servicebio, GB15165), FGF-2 (Servicebio, GB113970), KGF (bioss, BS-07434r), and EGF (Cell signaling, #9740) overnight in a wet box at 4 °C. Afterward, the slices were washed with PBS (0.1 м, pH = 7.4) in a rocker device for three times, and incubated with the corresponding goat anti-rabbit secondary antibody labeled with cyanine 3 at a room temperature for 50 min in dark condition. The results were observed by fluorescence microscope (Nikon Eclipse C1). All the relative fluorescence intensity data were processed by Image-Pro Plus 6.0.

For RNA-seq, total RNA samples from *P. aeruginosa*-infected wound tissues on day 8 post-therapy by the $NP^{M123/Fc}$+NIR-II group or by the Mock group were extracted, followed by library construction, Illumina sequencing, and data mining, as described previously.[11] A student *t*-test *P*-value < 0.05, and a foldchange > 2 were set as the statistical significance.

Cell Cytotoxicity Assay. NIH-3T3 cells (https://www.atcc.org/products/crl-1658) or IOSE-80 cells (https://www.cellosaurus.org/CVCL_5546) were cultured in Dulbecco's modified eagle medium (DMEM) in an incubator containing 5% CO_2 at 37°C. The cell suspension (1×10^4 cells per well) was seeded in 96-well plates and incubated for 24 h. $NP^{M123/Fc}$ was added into each of the well at different concentrations (0, 25, 50, 100 and 200 µg mL^{-1}, respectively). After 12 h incubation, the culture medium was discarded and CCK8 solution (100 µL) was added,

followed by further incubation for 4 h. CCK8 assay was applied to determine cell viability as described previously.[12]

Hemolysis Assay. To assess the hemolysis of $NP^{M123/Fc}$, blood from BALB/c mice was collected and then centrifuged for 10 min (3000 rpm) to obtain precipitated RBCs and resuspended in PBS. 3% RBCs solution was added into 1 mL PBS containing $NP^{M123/Fc}$ at 25, 50, 100 and 200 μg mL^{-1}, respectively (i.e., different experimental groups). The water and PBS groups were used as controls, respectively. All groups were incubated at room temperature for 4 h and then centrifuged for 10 min (3000 rpm) and photographed. Finally, each supernatant was obtained after centrifugation at 11000 rpm for 10 min and the absorbance at 550 nm was measured using a UV–Vis photo spectrometer. The hemolysis rate was calculated by using the following equation.

$$\text{Hemolysis rate }\% = \frac{\text{experimental group} - \text{negative control}}{\text{positive control} - \text{negative control}} \times 100\%$$

Statistical Analysis. All experiments were repeated at least 3 times and data was expressed as the mean or mean ± standard deviation (SD). Statistical significance was calculated using ordinary one-way ANOVA by GraphPad Prism 8.0. The asterisks signify statistically significant differences (ns: $P > 0.05$, $*P < 0.05$, $**P < 0.01$, $***P < 0.001$).

Animal Ethical Clearance. All animal experiment protocols were reviewed and approved by the Institutional Animal Care and Use Committee of Beijing Institute of Microbiology and Epidemiology (permit number: IACUC-DWZX-2023-047).

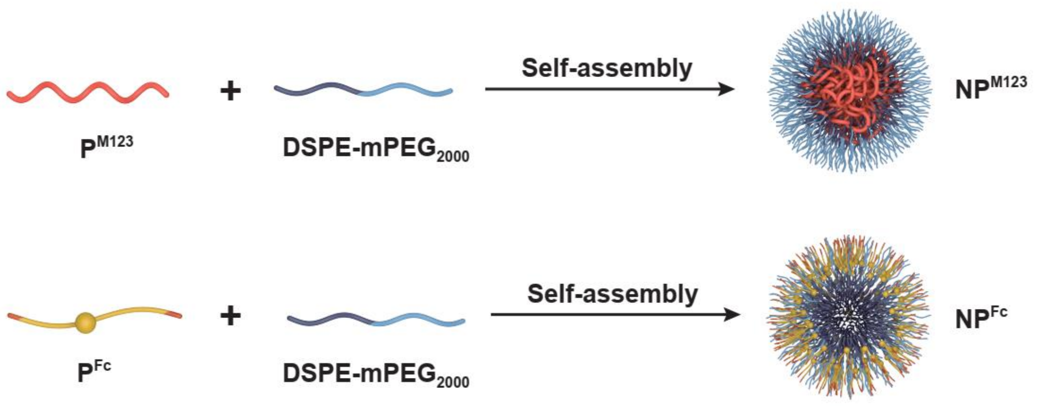

Scheme S1. Schematic illustration of preparation of NP^{M123} and NP^{Fc}.

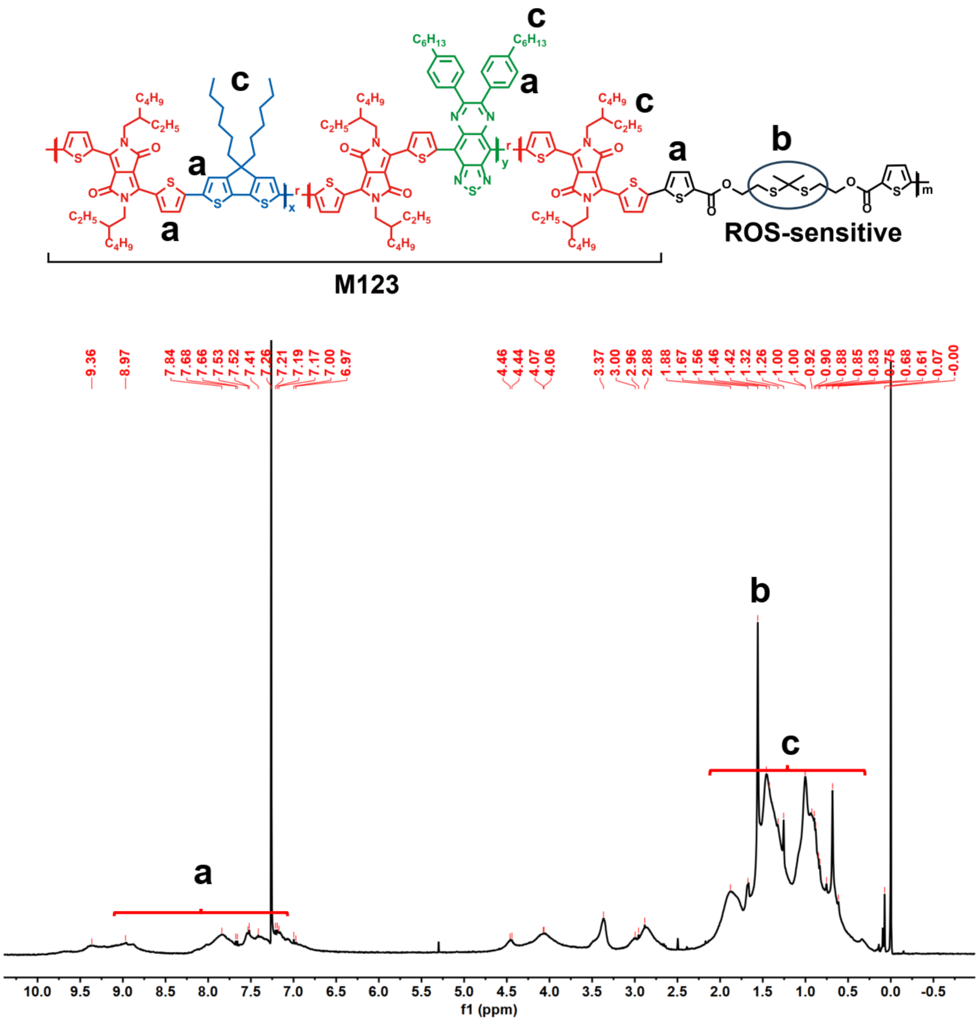

Figure S1. ^{1}H NMR spectrums of P^{M123}.

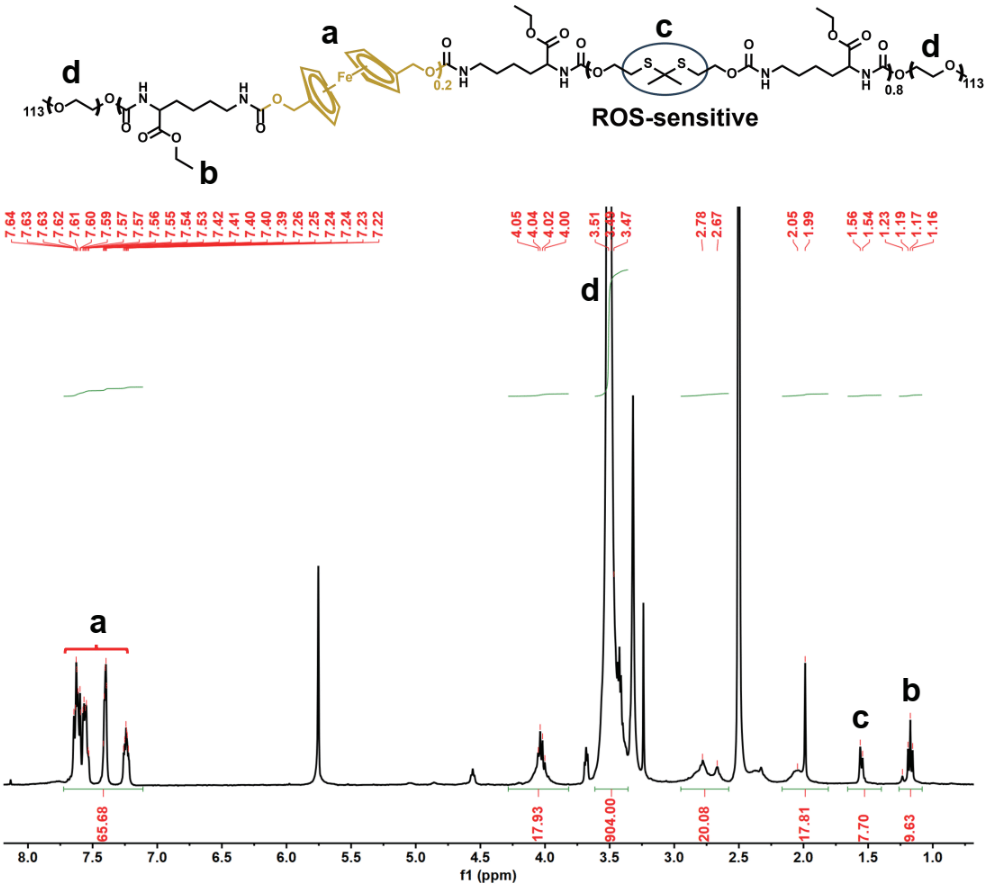

Figure S2. ^{1}H NMR spectrums of P^{Fc}.

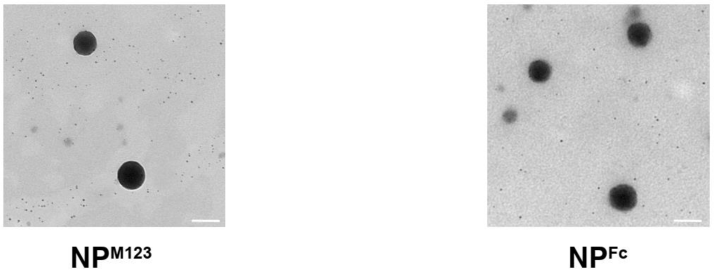

Figure S3. TEM images of NP^{M123} and NP^{Fc}. Scale bar is 100 nm.

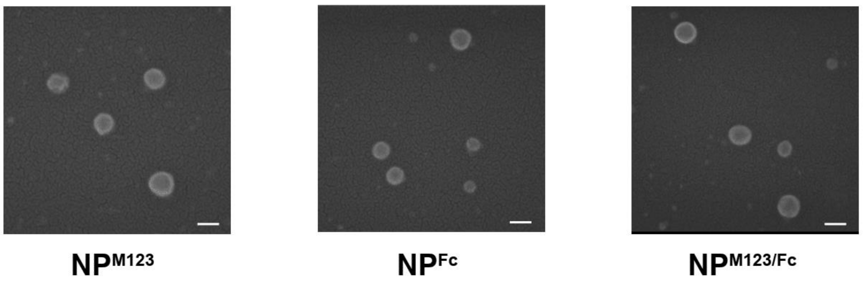

Figure S4. SEM images of NP^{M123}, NP^{Fc}, and $NP^{M123/Fc}$. Scale bar is 100 nm.

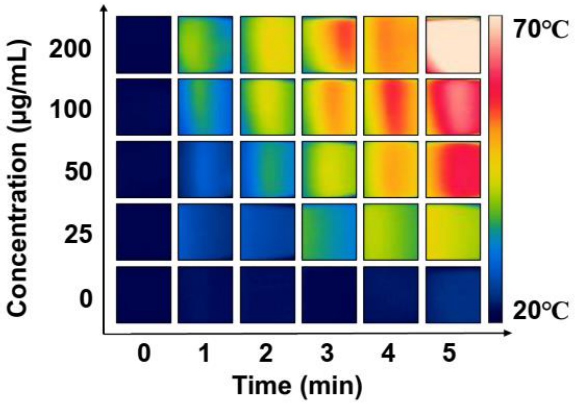

Figure S5. Thermal imaging for $NP^{M123/Fc}$ under 1064 nm laser irradiation (1.0 W cm^{-2}, 5 min).

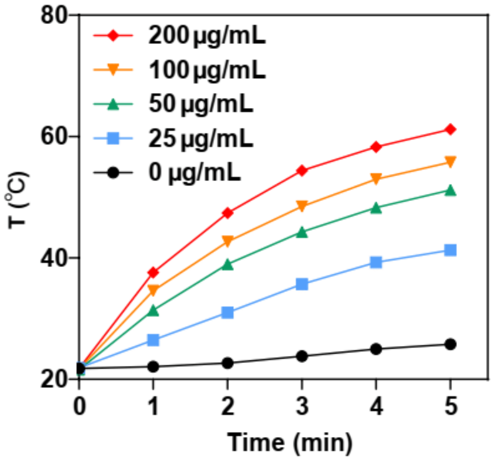

Figure S6. Time–concentration–temperature curves of NP^{M123} under 1064 nm laser irradiation (1.0 W cm^{-2}, 5 min).

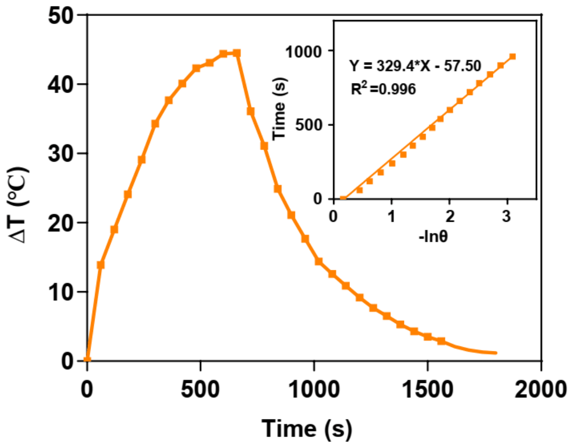

Figure S7. Time-dependent heating and cooling curve of 100 μg mL^{-1} NP$^{M123/Fc}$ under 1064 nm laser irradiation (1.0 W cm^{-2}, 11 min). The η value was calculated, through linear regression, from this heating and cooling curve.

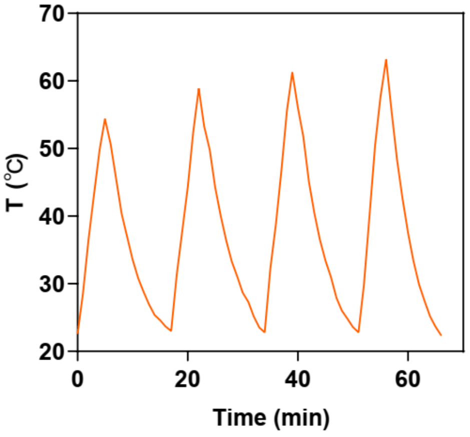

Figure S8. Time–temperature curve of 4 cycles of heating and cooling of NP^{M123} under 1064 nm laser irradiation (1.0 W cm^{-2}, 5 min).

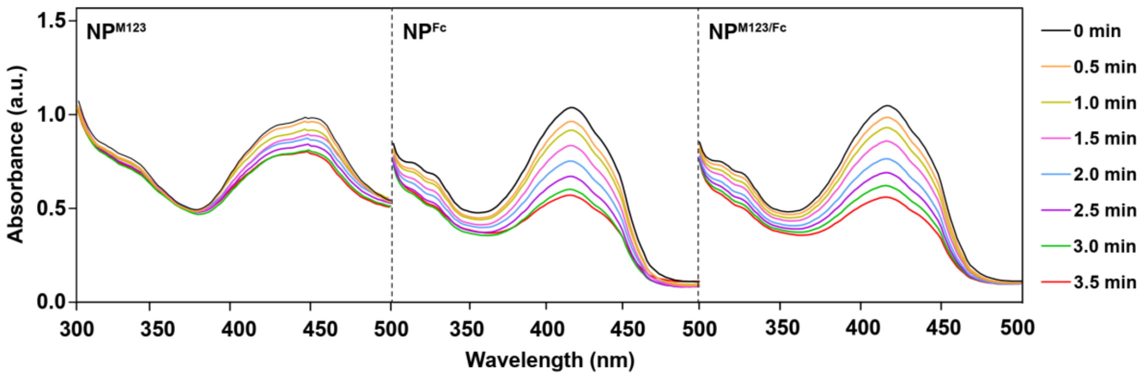

Figure S9. Absorption curves of DPBF as a selective trapping agent to detect ROS in the reaction mixture of NP^{M123}+H_2O_2, NP^{Fc}+H_2O_2, or NP$^{M123/Fc}$+H_2O_2 after different incubation times.

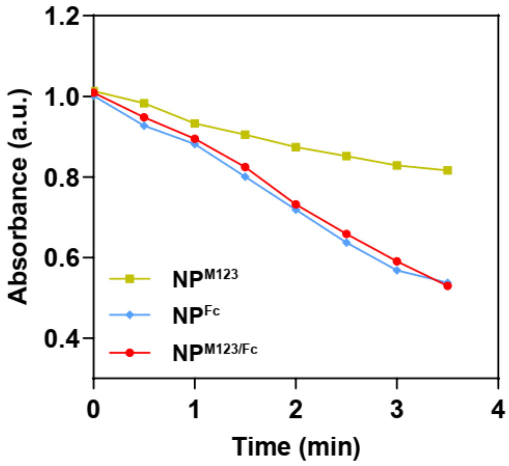

Figure S10. Trends of UV absorption at 410 nm as indicated by the above DPBF-based detection results.

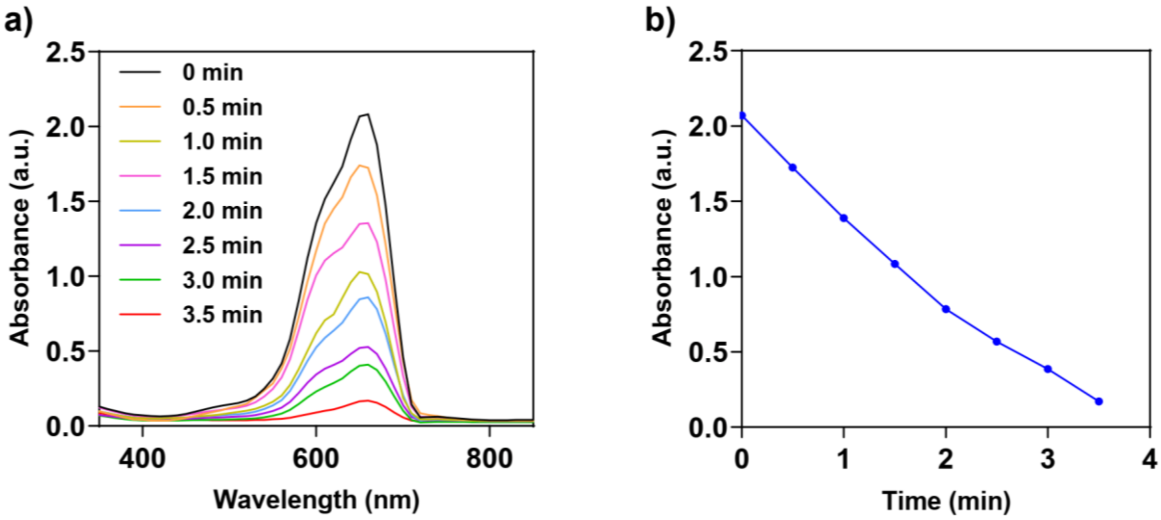

Figure S11. a) Absorption curves of MB as a selective trapping agent to detect •OH in the reaction mixture of NP^{Fc}+H_2O_2 after different incubation times. b) Trends of UV absorption at 410 nm as indicated by the above MB-based detection results.

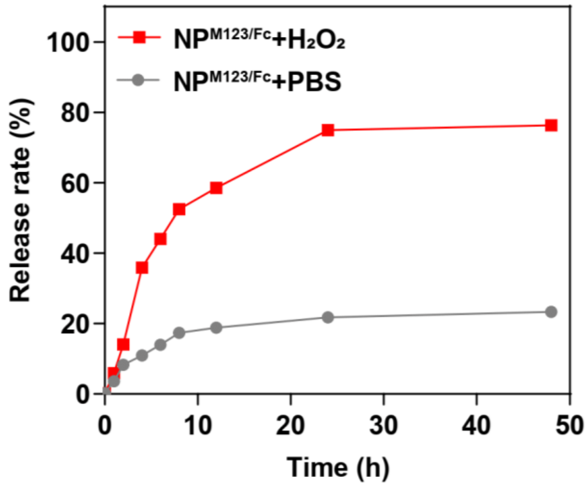

Figure S12. Fc release kinetics of NP$^{M123/Fc}$ *in vitro* was determined by ICP-MS.

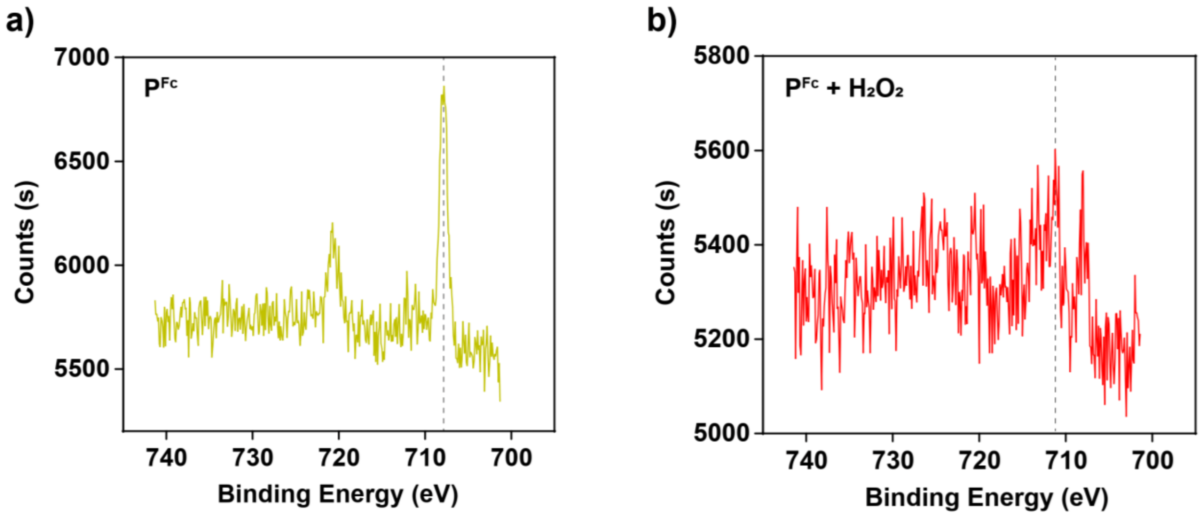

Figure S13. XPS spectra of P^{Fc}. a) In the absence of H_2O_2, the peaks at 707 Ev denote ferrous (+2) valence state. b) In the presence H_2O_2, the peaks at 711 Ev show ferric (+3) valence state.

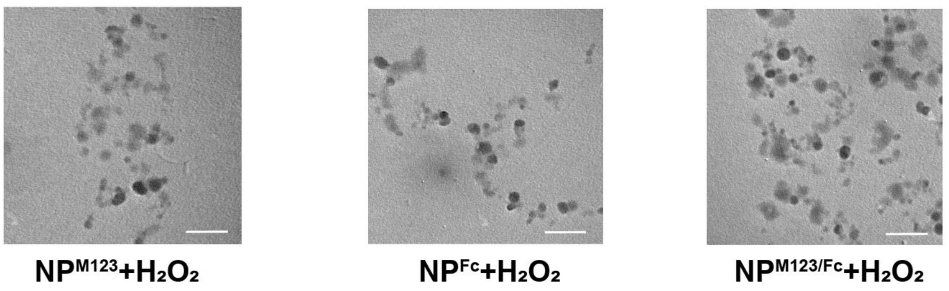

Figure S14. TEM images for NP^{M123}, NP^{Fc} and NP$^{M123/Fc}$ in the presence of H_2O_2. Scale bar is 100 nm.

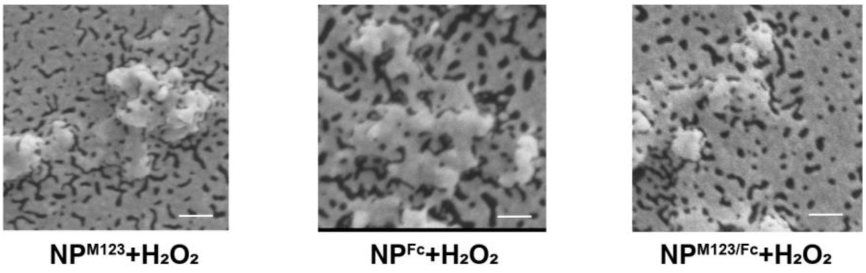

Figure S15. SEM images for NP^{M123}, NP^{Fc} and NP$^{M123/Fc}$ in the presence of H_2O_2. Scale bar is 100 nm.

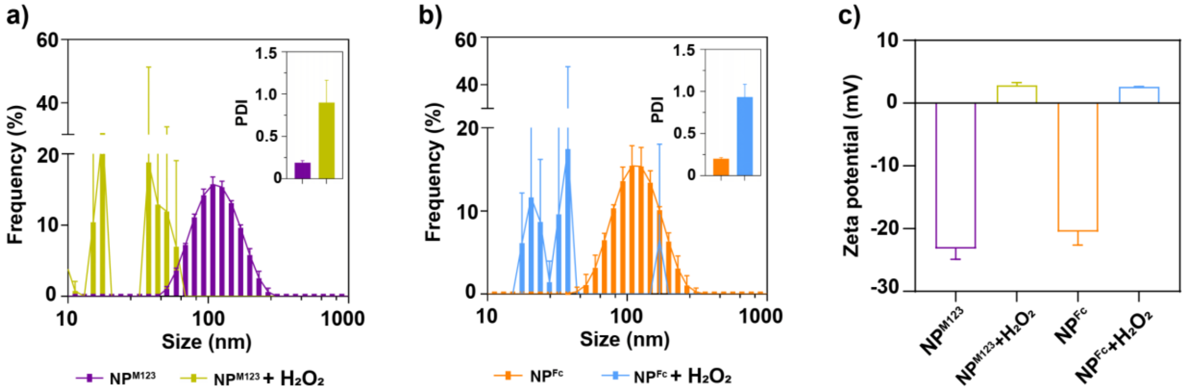

Figure S16. DLS measurement of diameter sizes and PDIs of a) NP^{M123} and b) NP^{Fc} in the presence or absence of H_2O_2. c) Average Zeta potentials of NP^{M123} and NP^{Fc} in the presence or absence of H_2O_2.

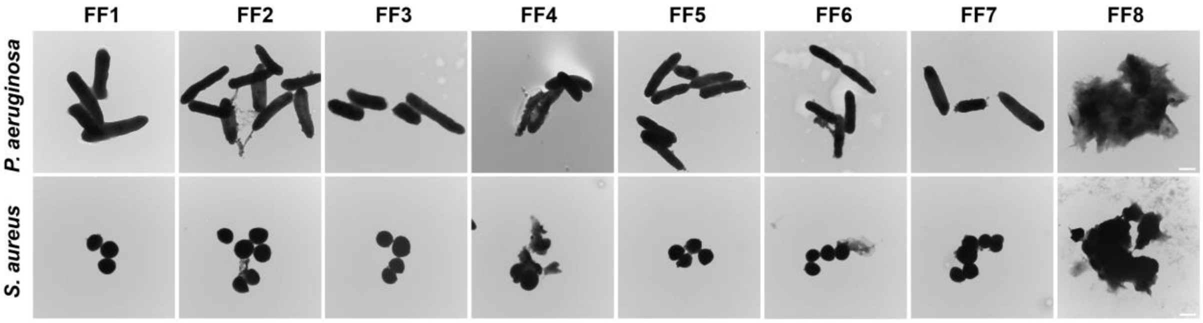

Figure S17. TEM images of *P. aeruginosa* and *S. aureus* post treatment. Scale bar is 500 nm. 1 mM H_2O_2 are added for all experiments. FF1: Mock, FF2: NIR-II, FF3: NP^{M123}, FF4: NP^{M123}+NIR-II, FF5: NP^{Fc}, FF6: NP^{Fc}+NIR-II, FF7: NP$^{M123/Fc}$, FF8: NP$^{M123/Fc}$+NIR-II. NIR-II represents 1064 nm laser irradiation (1.0 W cm^{-2}, 3.5 min).

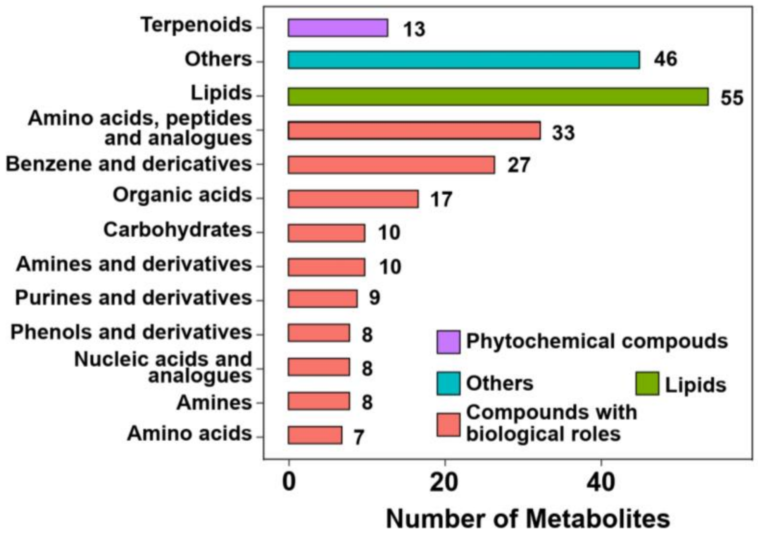

Figure S18. Classification of DRMs.

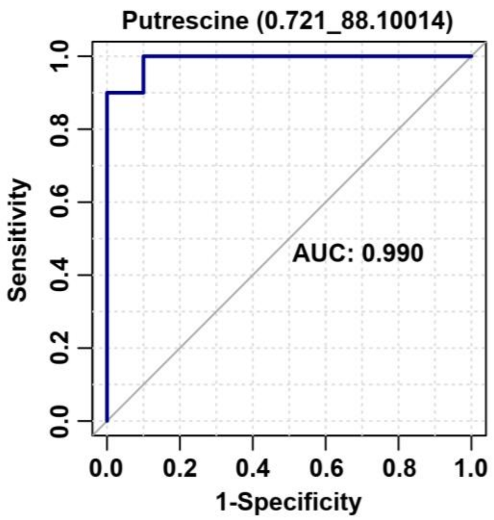

Figure S19. ROC curve for DRMs.

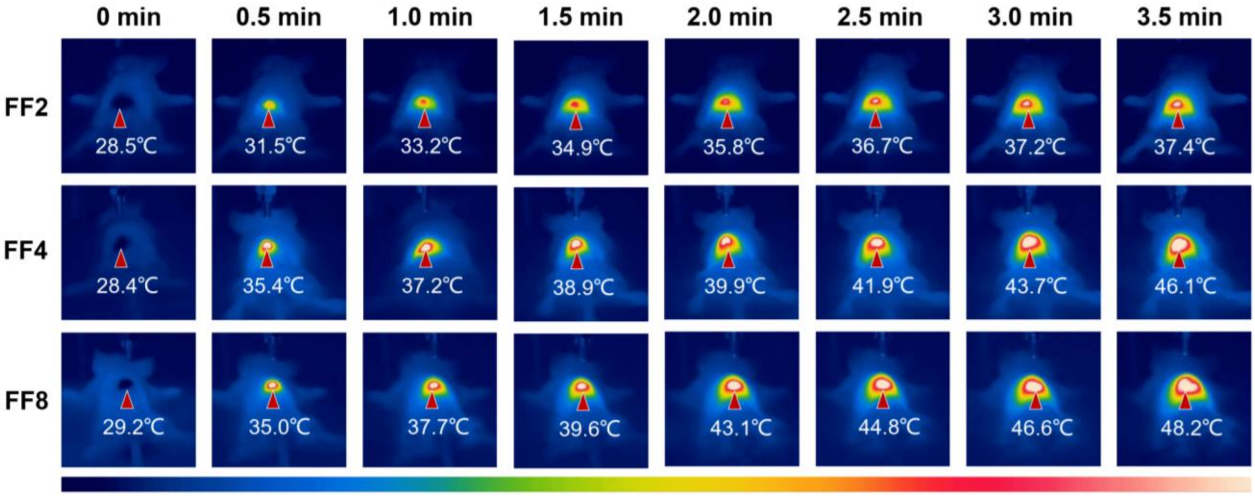

Figure S20. Time-dependent temperature increase at *P. aeruginosa*-infected wounds. FF2: NIR, FF4: NP^{M123}+NIR-II, FF8: $NP^{M123/Fc}$+NIR-II. NIR-II represents 1064 nm laser irradiation (1.0 W cm^{-2}, 3.5 min).

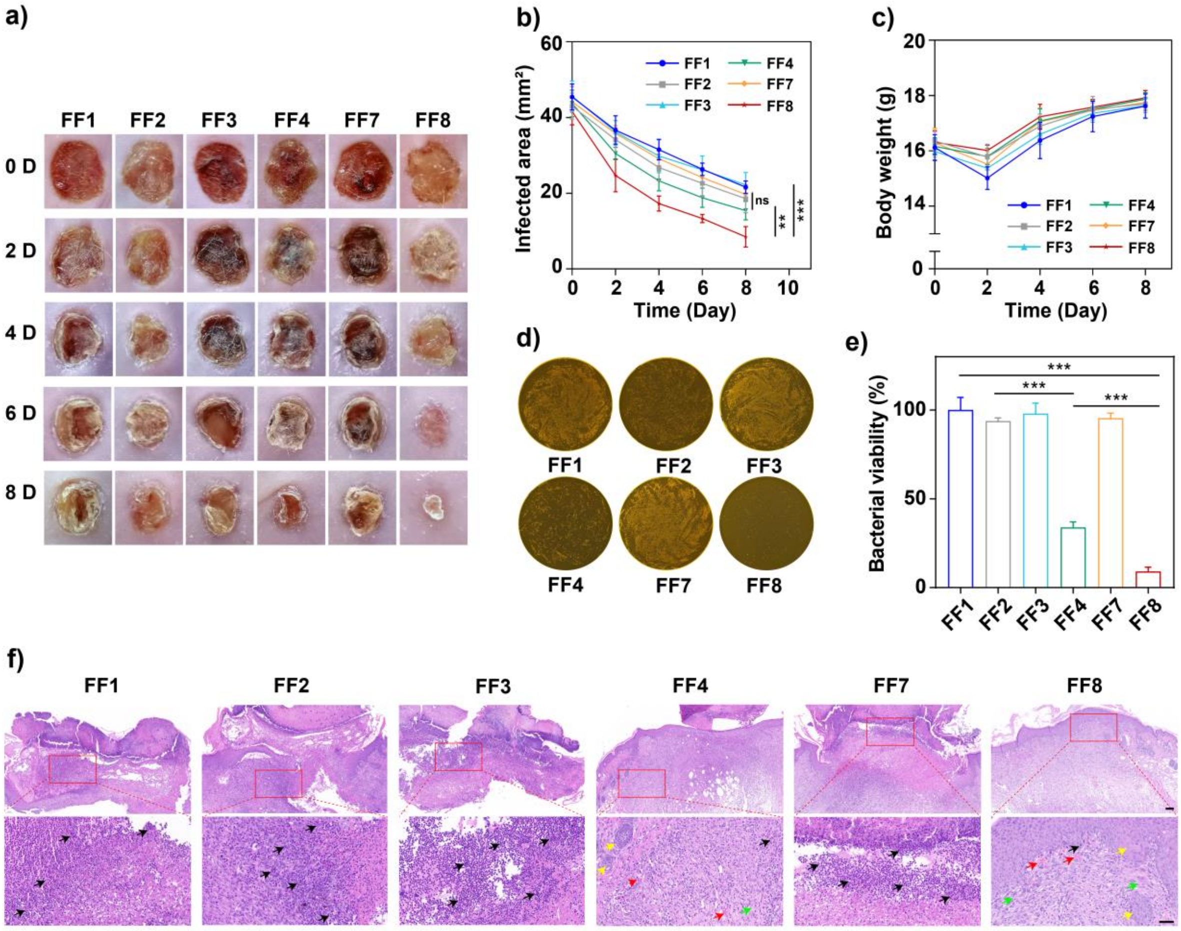

Figure S21. Therapeutic efficacy against *S. aureus*-infected wounds. FF1: Mock, FF2: NIR-II, FF3: NP^{M123}, FF4: NP^{M123}+NIR-II, FF7: $NP^{M123/Fc}$, FF8: $NP^{M123/Fc}$+NIR-II. NIR-II represents

1064 nm laser irradiation (1.0 W cm^{-2}, 3.5 min). a) Representative photos of infected wounds post therapy. Statistical analysis of b) wound areas and c) body weights post therapy. Data are presented as mean ± SD (n = 5). d) Representative photos of plate count agars for infected wounds on day 8 post-therapy, and e) corresponding statistical analysis of bacterial load counts. Data are presented as mean ± SD (n = 6). f) Representative photos of H&E staining of infected wounds (n = 3) on day 10 post-treatmnet. Scale bar is 100 µm. Black arrows denote inflammatory cells such as neutrophils, macrophages, and lymphocytes. Green arrows represent hair follicles. Yellow arrows show fibroblasts. Red arrows stand for new blood vessels. ns: $P \geq 0.05$, *: $P < 0.05$, **: $P < 0.01$, ***: $P < 0.001$.

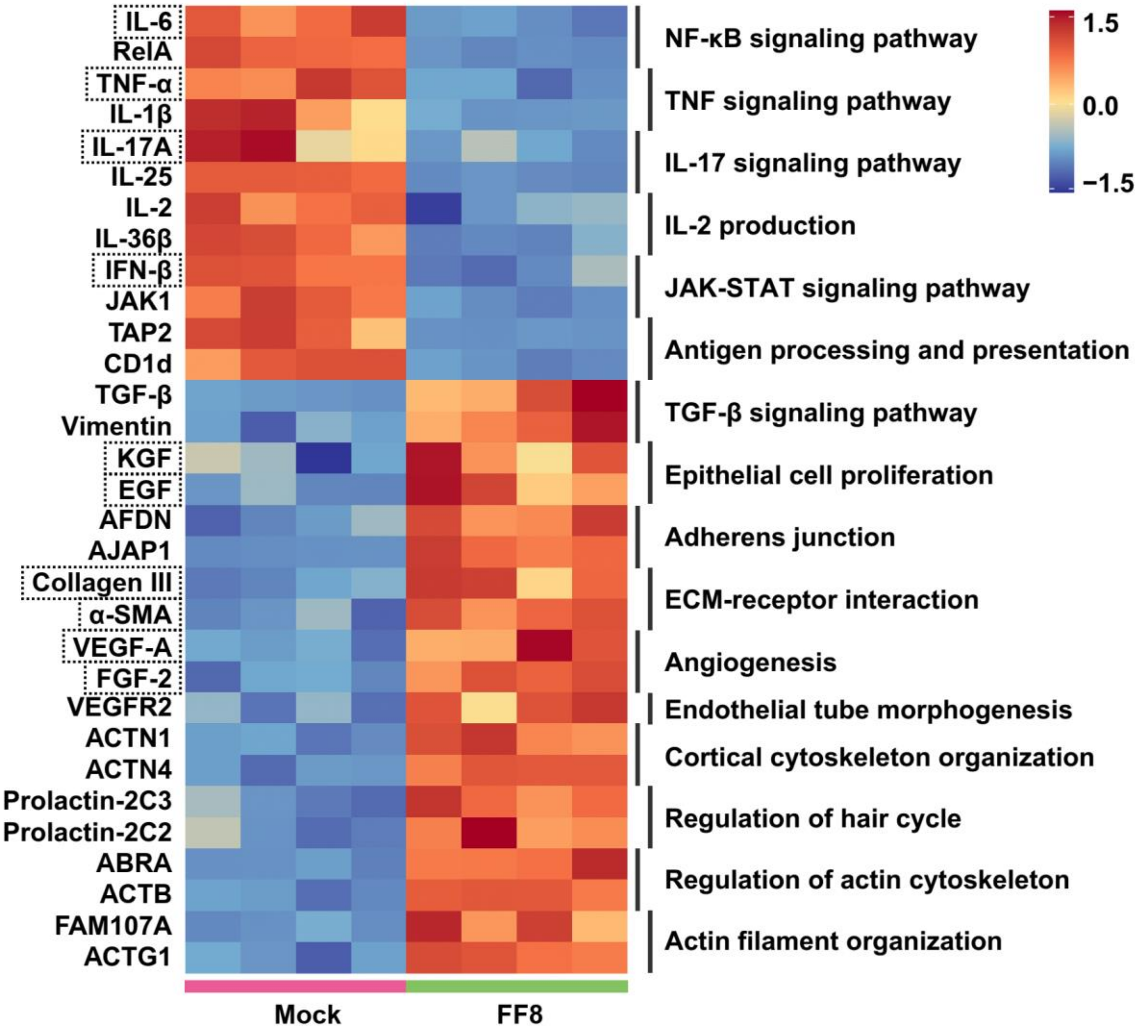

Figure S22. Heatmap of selected DRGs involved in proinflammation and wound healing genes. Boxes indicate the 10 DRGs revealed simultaneously by the immunohistochemistry and immunofluorescence staining assays and the RNA-seq assay.

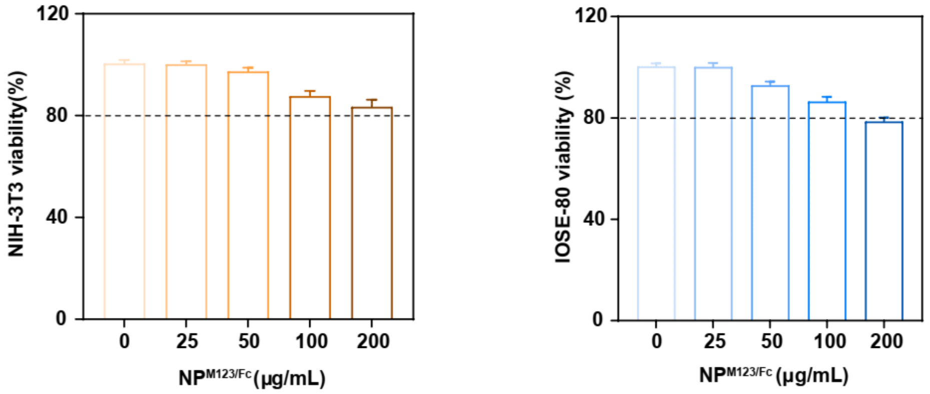

Figure S23. Viability of NIH-3T3 and IOSE-80 cells incubated with $NP^{M123/Fc}$ at different concentrations for 24 h. All data are presented as mean ± SD (n = 6).

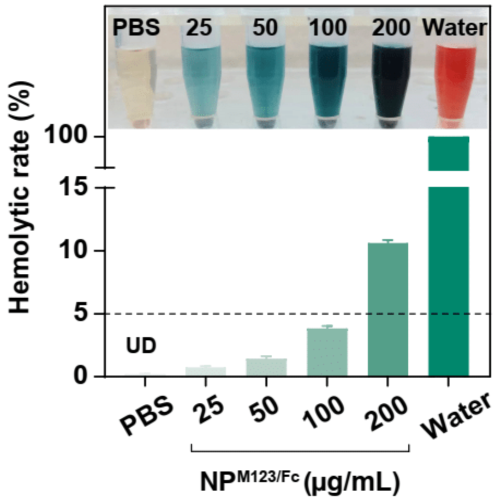

Figure S24. Hemolysis rates of RBCs incubated with different concentrations of $NP^{M123/Fc}$. The insert was the optical photograph. PBS and water were used as the negative and positive controls, respectively. All data are presented as mean ± SD (n = 3). UD: undetectable.

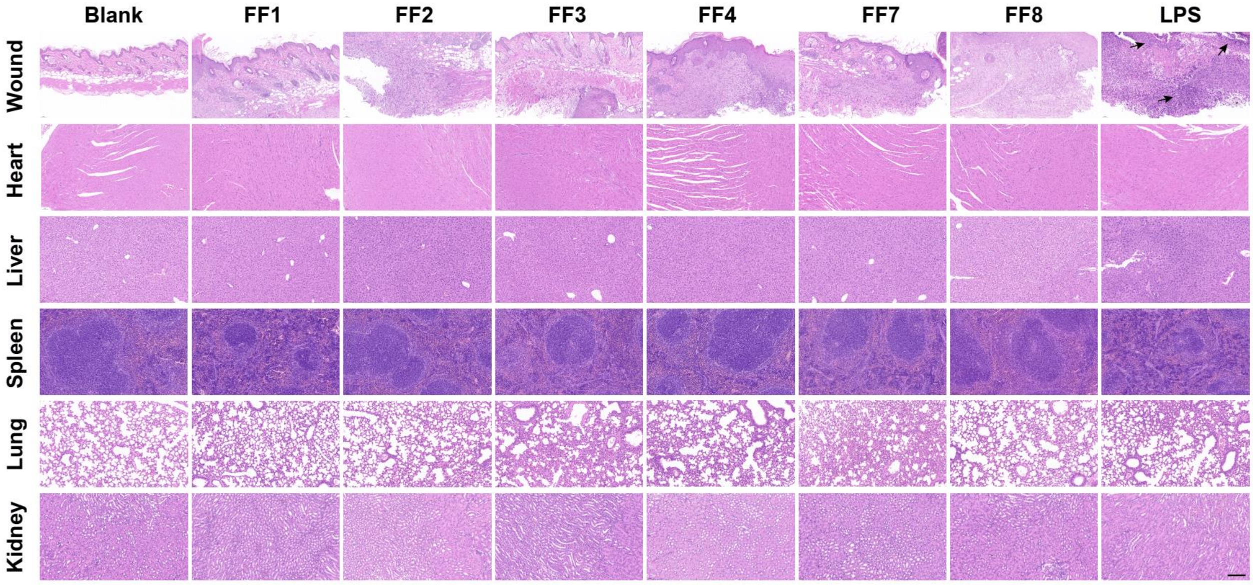

Figure S25. H&E staining of major organs in infection-free mice (n = 3) on day 8 post-treatment. Scale bar is 100 μm. Black arrows denote inflammatory cells such as neutrophils, macrophages, and lymphocytes. Blank represents wound-free skin tissue. FF1: Mock, FF2: NIR-II, FF3: NP^{M123}, FF4: NP^{M123}+NIR-II, FF7: $NP^{M123/Fc}$, FF8: $NP^{M123/Fc}$+NIR-II, LPS: 1 mg kg^{-1} LPS. NIR-II represents 1064 nm laser irradiation (1.0 W cm^{-2}, 3.5 min).

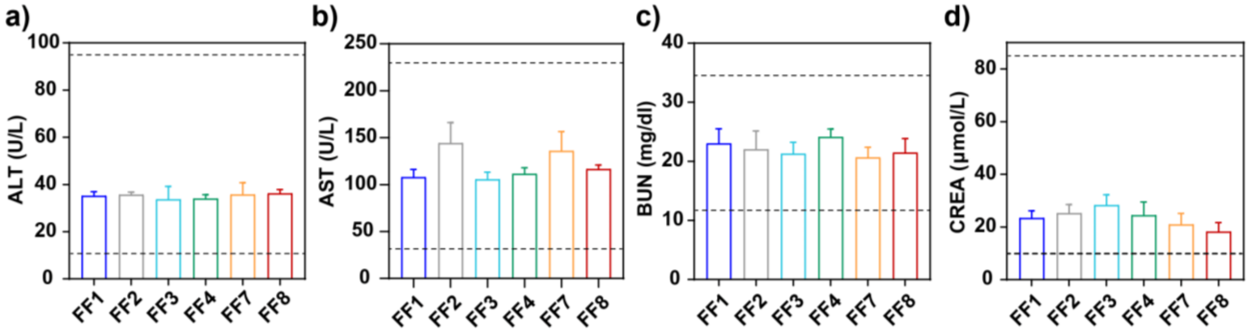

Figure S26. Detection of serum biochemical indicators including a) ALT (Reference range: 10.06-96.47 U L^{-1}), b) AST (Reference range: 36.31-235.48 U L^{-1}), c) BUN (Reference range: 10.81-34.74 mg dL^{-1}), and d) CREA (Reference range: 10.91-85.09 μmol L^{-1}) in infection-free mice (n = 3) on day 8 post-treatment. FF1: Mock, FF2: NIR-II, FF3: NP^{M123}, FF4: NP^{M123}+NIR-II, FF7: $NP^{M123/Fc}$, FF8: $NP^{M123/Fc}$+NIR-II. NIR-II represents 1064 nm laser irradiation (1.0 W cm^{-2}, 3.5 min).

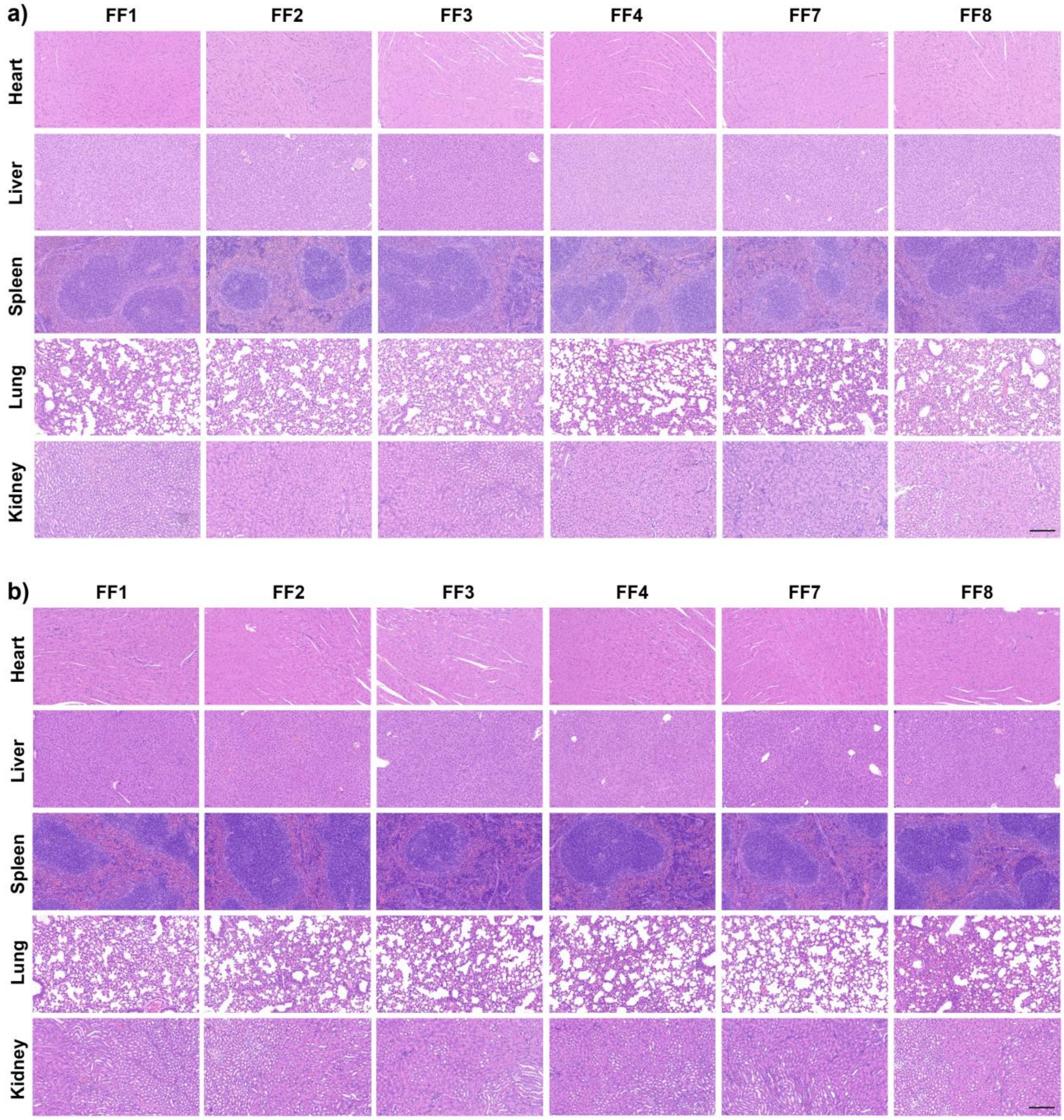

Figure S27. H&E staining of major organs in a) *P. aeruginosa*- and b) *S. aureus*-infected mice (n = 3) on day 10 post-therapy. Scale bar is 100 μm. FF1: Mock, FF2: NIR-II, FF3: NP^{M123}, FF4: NP^{M123}+NIR-II, FF7: $NP^{M123/Fc}$, FF8: $NP^{M123/Fc}$+NIR-II. NIR-II represents 1064 nm laser irradiation (1.0 W cm^{-2}, 3.5 min).

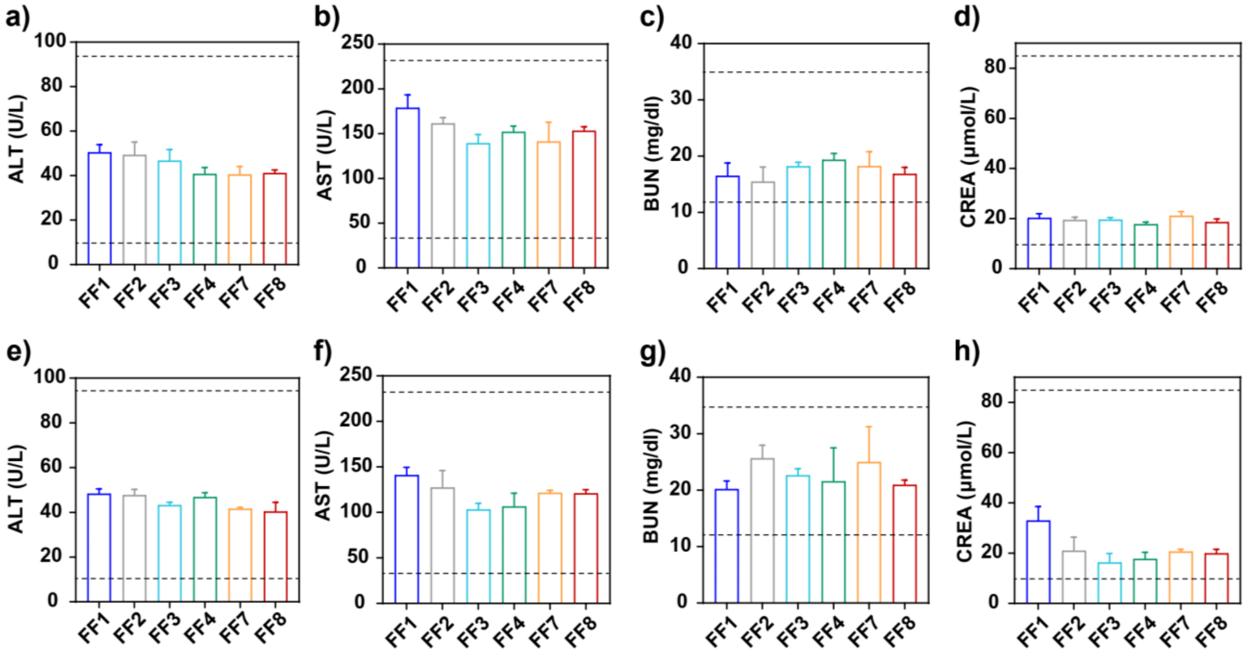

Figure S28. Detection of serum biochemical indicators a) ALT, b) AST, c) BUN, and d) CREA of *P. aeruginosa*-infected and e) ALT, b) AST, c) BUN, and d) CREA in *S. aureus*-infected mice (n = 3) on day 10 post-therapy. FF1: Mock, FF2: NIR-II, FF3: NP^{M123}, FF4: NP^{M123}+NIR-II, FF7: $NP^{M123/Fc}$, FF8: $NP^{M123/Fc}$+NIR-II. NIR-II represents 1064 nm laser irradiation (1.0 W cm^{-2}, 3.5 min).

Reference

[1] H. Zhou, D. Tang, X. Kang, H. Yuan, Y. Yu, X. Xiong, N. Wu, F. Chen, X. Wang, H. Xiao, D. Zhou, *Adv. Sci.* **2022**, 9, e2200732.

[2] Y. Gao, H. Zhang, L. Tang, F. Li, L. Yang, H. Xiao, J. Karges, W. Huang, W. Zhang, C. Liu, *Adv. Sci.* **2023**, DOI: 10.1002/advs.202300806e2300806.

[3] D. Xi, M. Xiao, J. Cao, L. Zhao, N. Xu, S. Long, J. Fan, K. Shao, W. Sun, X. Yan, X. Peng, *Adv. Mater.* **2020**, 32, e1907855.

[4] X. Pan, N. Wu, S. Tian, J. Guo, C. Wang, Y. Sun, Z. Huang, F. Chen, Q. Wu, Y. Jing, *Adv. Funct. Mater.* **2022**, 32, 2112145.

[5] B. A. Diep, S. R. Gill, R. F. Chang, T. H. Phan, J. H. Chen, M. G. Davidson, F. Lin, J. Lin, H. A. Carleton, E. F. Mongodin, G. F. Sensabaugh, F. Perdreau-Remington, *Lancet* **2006**, 367, 731.

[6] C. M. Hudson, Z. W. Bent, R. J. Meagher, K. P. Williams, *PLoS One* **2014**, 9, e99209.

[7] H. Y. Ou, S. N. Kuang, X. He, B. M. Molgora, P. J. Ewing, Z. Deng, M. Osby, W. Chen, H. H. Xu, *Sci. Rep.* **2015**, 5, 8643.

[8] S. Chen, S. Zhao, X. Wang, L. Zhang, E. Jiang, Y. Gu, A. J. Shangguan, H. Zhao, T. Lv, Z. Yu, *Transl. Lung Cancer Res.* **2015**, 4, 775.

[9] Y. Liu, P. Maccarini, G. M. Palmer, W. Etienne, Y. Zhao, C. T. Lee, X. Ma, B. A. Inman, T. Vo-Dinh, *Sci. Rep.* **2017**, 7, 8606.

[10] H. Wang, S. Wang, R. Wang, X. Wang, K. Jiang, C. Xie, C. Zhan, H. Wang, W. Lu, *Nanoscale* **2019**, 11, 13069.

[11] X. OuYang, X. Xu, Q. Qin, C. Dai, H. Wang, S. Liu, L. Hu, X. Xiong, H. Liu, D. Zhou, *Adv. Mater.* **2023**, 35, e2304514.

[12] L. Belyanskaya, P. Manser, P. Spohn, A. Bruinink, P. Wick, *Carbon* **2007**, 45, 2643.
